# The infant gut resistome is associated with *E. coli* and early-life exposures

**DOI:** 10.1186/s12866-021-02129-x

**Published:** 2021-07-02

**Authors:** Rebecca M. Lebeaux, Modupe O. Coker, Erika F. Dade, Thomas J. Palys, Hilary G. Morrison, Benjamin D. Ross, Emily R. Baker, Margaret R. Karagas, Juliette C. Madan, Anne G. Hoen

**Affiliations:** 1 Department of Epidemiology, Geisel School of Medicine at Dartmouth, Hanover, NH USA; 2 Department of Microbiology & Immunology, Geisel School of Medicine at Dartmouth, Hanover, NH USA; 3 Oral Biology Department, Rutgers School of Dental Medicine, Newark, NJ USA; 4 Center for Molecular Epidemiology, Geisel School of Medicine at Dartmouth, Hanover, NH USA; 5 Marine Biological Laboratory, Woods Hole, MA USA; 6 Department of Orthopaedics Geisel School of Medicine at Dartmouth , NH Hanover, USA; 7 Department of Pediatrics, Children’s Hospital at Dartmouth, Lebanon, NH USA; 8 Children’s Environmental Health & Disease Prevention Research Center at Dartmouth, Hanover, NH USA; 9 Department of Biomedical Data Science, Geisel School of Medicine at Dartmouth, Hanover, NH USA

**Keywords:** Gastrointestinal microbiome, Antibiotic resistance, Epidemiology, Cohort studies, Infants

## Abstract

**Background:**

The human gut microbiome harbors a collection of bacterial antimicrobial resistance genes (ARGs) known as the resistome. The factors associated with establishment of the resistome in early life are not well understood. We investigated the early-life exposures and taxonomic signatures associated with resistome development over the first year of life in a large, prospective cohort in the United States. Shotgun metagenomic sequencing was used to profile both microbial composition and ARGs in stool samples collected at 6 weeks and 1 year of age from infants enrolled in the New Hampshire Birth Cohort Study. Negative binomial regression and statistical modeling were used to examine infant factors such as sex, delivery mode, feeding method, gestational age, antibiotic exposure, and infant gut microbiome composition in relation to the diversity and relative abundance of ARGs.

**Results:**

Metagenomic sequencing was performed on paired samples from 195 full term (at least 37 weeks’ gestation) and 15 late preterm (33–36 weeks’ gestation) infants. 6-week samples compared to 1-year samples had 4.37 times (95% CI: 3.54–5.39) the rate of harboring ARGs. The majority of ARGs that were at a greater relative abundance at 6 weeks (chi-squared *p* < 0.01) worked through the mechanism of antibiotic efflux. The overall relative abundance of the resistome was strongly correlated with Proteobacteria (Spearman correlation = 78.9%) and specifically *Escherichia coli* (62.2%) relative abundance in the gut microbiome. Among infant characteristics, delivery mode was most strongly associated with the diversity and relative abundance of ARGs. Infants born via cesarean delivery had a trend towards a higher risk of harboring unique ARGs [relative risk = 1.12 (95% CI: 0.97–1.29)] as well as having an increased risk for overall ARG relative abundance [relative risk = 1.43 (95% CI: 1.12–1.84)] at 1 year compared to infants born vaginally.

**Conclusions:**

Our findings suggest that the developing infant gut resistome may be alterable by early-life exposures. Establishing the extent to which infant characteristics and early-life exposures impact the resistome can ultimately lead to interventions that decrease the transmission of ARGs and thus the risk of antibiotic resistant infections.

**Supplementary Information:**

The online version contains supplementary material available at 10.1186/s12866-021-02129-x.

## Background

The overuse of antibiotics in medicine and agriculture has contributed to the growing public health burden of antimicrobial resistance [1]. Studies have identified sets of antimicrobial resistance genes (ARGs) known collectively as the resistome [2, 3] in animals, humans, and the environment [4–6]. The human gut microbiome is a critical reservoir of ARGs [7, 8], but differential acquisition and composition of ARGs in the beginning of life is still largely unexplored.

Infants and young children are prescribed more antibiotics than any other age group [9, 10]. Multiple studies have focused on understanding the role of early-life antibiotic exposures to the infant gut resistome noting enrichment for ARGs that did and did not confer resistance to the antibiotic prescribed [11–14]. Moreover, ARGs have been identified in infants’ first stools following delivery and prior to any direct antibiotic exposure [15–17]. The abundance of ARGs in the infant gut has been shown to decrease with age [11, 15, 18, 19]. These findings have motivated studies to explore early-drivers that shape the resistome beyond infant antibiotic usage. Factors that have specifically been assessed in relation to the infant gut resistome include age of the infant [11, 18, 19], type of delivery [15, 20], breast versus formula feeding [21], gestational age [11, 13, 21], and intrapartum antibiotic usage [19]. Prior investigations assessing how early-life factors impact the resistome have focused on preterm infants [11, 13, 22] or were based on small cohorts (< 50) of full term infants [8, 12, 14, 17–19]. The two largest studies in full-term infants [15, 20] reported on the count and types of ARGs but did not examine longitudinal changes in ARGs or early-life factors associated with resistome development. This has led to a gap in understanding how early-life factors shape the resistome of full term infants.

Resistomic and taxonomic composition are intrinsically intertwined, but the extent to which early-life factors shape the resistome via infant gut microbial composition has not been established. Variation to ecological successional patterns of microbiome assembly has been observed by our group and others in association with gestational age [13], delivery mode [15, 20, 23, 24], feeding mode [23, 25], intrapartum antibiotic exposure [13, 19, 20, 26–29], geographic diversity [30, 31], and antibiotic usage [11, 12, 30, 32]. As ARGs can be passed horizontally between bacteria through mobile genetic elements or vertically within the infant gut [11, 12, 19] and differential infant characteristics impact the trajectory of microbial composition [12, 15, 33], taxonomic composition is an important factor to consider in context to resistome development.

Despite the wide number of studies that have investigated the resistome, no study has simultaneously evaluated the independent effects of differential infant exposures and microbiome taxonomic composition on the resistome in a general population of infants. Our study objectives were to fill two important gaps in the epidemiology of human resistome development. First, we aimed to establish the baseline composition of the resistome at approximately 6 weeks and 1 year of life in a general population United States birth cohort. Second, we aimed to assess how early-life exposures, in conjunction and independently to microbial composition, affect the infant gut resistome. We found that infant gut microbiota harbored a greater relative abundance of ARGs at 6 weeks than 1 year. A similar number of unique ARGs were found at each time point, but the types of ARGs varied considerably. Overall, differential resistome composition was impacted by early-life exposures including delivery mode, but was primarily driven by taxonomic composition during the first year of life.

## Results

### Sample selection and baseline characteristics

We performed whole metagenome sequencing on stool samples from infants enrolled in the New Hampshire Birth Cohort Study (NHBCS) to profile the gut resistome and microbiome. Our study consisted of infants born between 2012 and 2017 who had a stool sample collected at approximately 6 weeks and 12 months of life. From 238 infants with paired 6-week and 1-year samples, we excluded infants if one or both samples had missing sample age, feeding mode, or intrapartum antibiotic exposure data. This resulted in a total of 420 stool samples from 210 infants. Of these, 195 (92.9%) infants were born full term (at least 37 weeks’ gestation) and 15 (7.1%) were born late preterm (33–36 weeks’ gestation) (Table 1). Infants commonly were vaginally delivered (*n* = 152; 72.4%) and slightly more than half were exposed to intrapartum antibiotics (*n* = 115; 54.8%). The majority of infants who were vaginally delivered and received intrapartum antibiotics (*n* = 63) were given penicillin-like antibiotics (*n* = 41; 65%) while the majority of women receiving intrapartum antibiotics for a cesarean birth (*n* = 52) received cephalosporins (*n* = 34; 65%). Most infants (93.8%) were ethnically of European Ancestry and White race which reflects the underlying study population in the surrounding rural northern New England community. No covariate analyzed was associated with analysis using single or paired-end reads (Additional File 2: **Table S1**).

**Table 1:** Baseline Characteristics of Paired Infant Samples (*n* = 210)

**Infant Characteristics**	
Infant Sex (%)	
Female	89 (42.4)
Male	121 (57.6)
Infant Race (%)	
White	197 (93.8)
Other	13 (6.2)
Feeding Mode at 6-Week Sample (%)	
Breast fed	157 (74.8)
Formula fed	7 (3.3)
Combination	46 (21.9)
Feeding Mode at 1-Year Sample (%)	
Breastfed	70 (33.3)
Formula fed	7 (3.3)
Combination	133 (63.3)
Antibiotics During Initial Hospitalization (%)	
No	203 (96.7)
Yes	7 (3.3)
Gestational Age at Birth in Weeks [Mean (SD)]	39.05 (1.56)
Birth Weight in Grams [Mean (SD)]	3414 (507)
Age at 6-Week Sample Collection in Days [Mean (SD)]	46.78 (18.44)
Age at 1-Year Sample Collection in Days [Mean (SD)]	375.32 (35.69)
**Maternal Characteristics**	
Delivery Mode (%)	
Vaginal	152 (72.4)
Cesarean section	58 (27.6)
Prenatal Antibiotics Prior to Delivery (%)	
No	157 (74.8)
Yes	39 (18.6)
Missing	14 (6.7)
Group B *Streptococcus* Positive (%)	
No	137 (65.2)
Yes	68 (32.4)
Missing	5 (2.4)
Parity (%)*	
Nulliparous	104 (49.8)
One	72 (34.4)
At least two	33 (15.8)
Intrapartum Antibiotic Exposure Class** (%)	
None	95 (45.2)
Penicillin	43 (20.5)
Cephalosporin	43 (20.5)
Multiple	25 (11.9)
Other	4 (1.9)

*One mother was missing parity status

**Infants were grouped according to intrapartum antibiotic exposures using the following categories: no antibiotics; penicillin-like antibiotics only (amoxicillins, penicillins); cephalosporins only (cefazolin, cephalexin); multi-drug classes (two or more antibiotics characterized as penicillin, cephalosporin, vancomycin, clindamycin, and/or gentamicin); or “other” antibiotics such as aminoglycosides, glycopeptides, or lincomycin

### Descriptive overview of the resistome and microbiome

Using ShortBRED [34], we profiled ARGs using markers for ARGs derived from shortened proteins stored in the Comprehensive Antibiotic Resistance Database (CARD) [35]. Our primary outcomes were the number of unique ARGs and the overall relative abundance of ARGs in reads per kilobase of reference sequence per million sample reads (RPKM). The prevalence, mean, and median values for all ARGs profiled (*n* = 887) were calculated across all samples and among samples with the gene present (Additional File 2: **Table S2)**. **Table S2** also includes information on the predicted pathogenic species origin, resistance mechanism, and associated drug classes of each ARG marker. 359 different ARG markers were identified in the 420 samples and 241 were identified in at least 1% of samples. There was a median of 77 (interquartile range: 45–94) unique ARG markers per sample and all samples had at least 7 unique ARG markers (Additional File 1: **Fig. S1a**). Total median (interquartile range) relative abundance of ARG markers was 1086 (570–2459) RPKM.

**Fig. 1:**
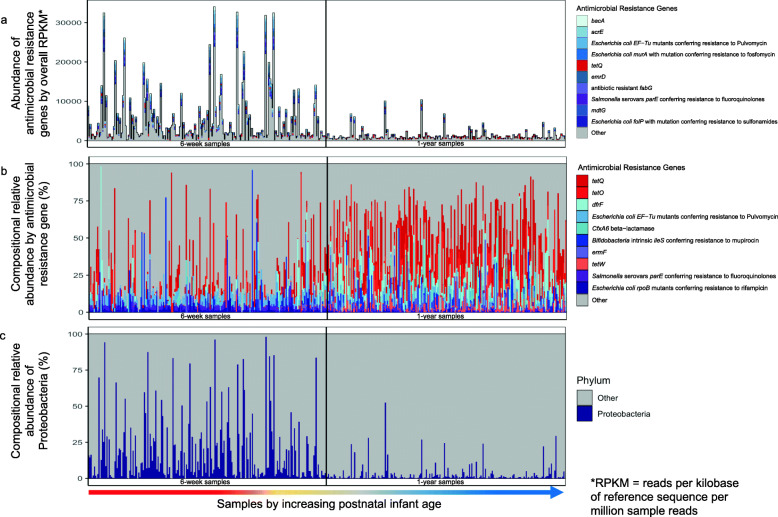
Composition of the resistome and microbiome in 420 infant gut samples. All samples are ordered by increasing sample age at collection and the black vertical lines demarcate the 6-week samples from the 1-year samples. (**a**) Overall relative abundance (in RPKM) of the 10 antimicrobial resistance genes with the greatest mean abundance across all samples. (**b**) Compositional relative abundance of the 10 antimicrobial resistance genes with the greatest mean compositional abundance across all samples. (**c**) Compositional relative abundance of Proteobacteria. For (**a**) and (**b**), antimicrobial resistance genes are colored red if they are *tet* genes and blue or purple otherwise

Resistome relative abundance and composition differed in the 6-week and 1-year samples. The overall relative abundance of ARGs was significantly higher at 6 weeks than 1 year (Fig. 1**a;** Additional File 1: **Fig. S1b**). Many differentially abundant ARG markers by postnatal age of the infant were related to antibiotic efflux (Additional File 1: **Fig. S2**). The most commonly identified (94.3% prevalence) ARG marker for 6-week samples was *Enterobacter cloacae acrA* [Antibiotic Resistance Ontology (ARO): 3004042], a resistance-nodulation-cell-division antibiotic efflux pump. *tetO* (ARO: 3000190) was most commonly found in 1-year samples (97.6%). Through assessing the resistome compositionally, we identified that 6-week samples had a more even dispersion of unique ARGs while 1-year samples mainly were composed of 1 or 2 dominant ARGs (Fig. 1**b**). Specifically, 1-year samples had a high relative abundance of either *tetO*, *tetW* (ARO: 3000194), or *tetQ* (ARO: 3000191) which confer resistance to tetracycline antibiotics, along with *dfrF* (ARO: 3002867) which confers resistance to diaminopyrimidine antibiotics and *CfxA6* (ARO: 3003097) a beta-lactamase gene conferring resistance to cephamycin. The overall relative abundance in RPKM of ARGs conferring resistance to tetracycline, however, was greater in 6-week samples as compared to 1-year samples. The predicted pathogenic resistome origins for *tetO* and *tetW* are from a variety of gram-positive and negative bacteria including *Enterococcus* spp. and *Klebsiella* spp. *tetQ*’s predicted resistome origin via CARD is only from *Acinetobacter baumannii* and *Enterobacter hormaechei*. However, previous research has found *tetQ* in a variety of other gram-positive and negative organisms, but is commonly identified in *Prevotella* [36] and *Bacteroides* [36, 37].

To determine the influence of microbial composition on resistome composition, we taxonomically profiled the infant stool samples using MetaPhlAn2 [38]. Previous research has identified Proteobacteria and *E. coli* relative abundance to be associated with ARG relative abundance [13, 15, 19, 39, 40]. Likewise, certain ARGs that we also profiled including *acrF*, *mdth*, *mdtE*, *tolC*, and *acRb* have previously been associated with *Escherichia*/*Shigella* in infants [14]. Thus, based on prior research and visually identifying Proteobacteria to be correlated with the relative abundance of the resistome (Fig. 1**c**), we focused on this taxon as a potential mediator between infant characteristics and resistome composition.

### The resistome is driven by early-life factors

After profiling resistomic and taxonomic composition, we aimed to explore which covariates and early-life factors may impact resistome development. We used negative binomial regression to assess how delivery mode, feeding mode, intrapartum antibiotic exposure, sex, and initial antibiotic exposure during hospitalization, controlling for log_10_-number of reads, impacted the count of unique ARG markers (Additional File 2: **Table S3**). Sample age (6-week vs. 1-year) was not associated with unique ARGs (relative risk = 0.99; 95% CI: 0.9–1.08). Nonetheless, we stratified the remainder of our results because we hypothesized that, since different types of ARGs were present at 6 weeks and 1 year, covariates and early-life factors may be differentially associated at each time point. Cesarean delivery, as compared to vaginal delivery, was associated with a differential risk of having unique ARGs but in different directions at 6 weeks and 1 year. Cesarean section delivered infants had a decreased risk [0.84 (95% CI: 0.72–0.99)] at 6 weeks and yet an increased risk [1.12 (95% CI: 0.97–1.29)] at 1 year of having unique ARGs compared to vaginally delivered infants. Compared to exclusive breastfeeding at 6 weeks, any formula feeding exposure was associated with an increased risk of having diverse ARGs [relative risk = 1.29 (95% CI: 0.92–1.89) for infants formula fed exclusively and relative risk = 1.13 (95% CI: 0.97–1.33) for combination fed infants] with the opposing trend at 1 year [relative risk = 0.74 (95% CI: 0.54–1.04) for infants formula fed exclusively; relative risk = 0.81 (95% CI: 0.72–0.92) for combination fed infants]. Although males had no differential risk of harboring a unique number of ARGs at 6 weeks compared to females, at 1 year males had a decreased risk [relative risk = 0.84 (95% CI 0.75–0.95)] of harboring unique ARGs.

In addition to profiling how covariates and early-life factors may contribute to different types of ARGs, we also assessed how these factors impacted the overall relative abundance of the resistome. In negative binomial regression analyses assessing total RPKM of ARG markers, we found that 6-week samples harbored a higher relative abundance of ARGs compared to 1-year samples. After adjustment for delivery mode, feeding mode, initial antibiotic exposure after hospitalization, intrapartum antibiotic exposure, gestational age, and sex, 6-week samples, as compared to 1 year samples, had 4.37 times (95% CI: 3.54–5.39) the rate of harboring ARG markers (Additional File 2: **Table S4**). At 6 weeks, male infants had 1.41 times (95% CI: 1.02–1.94) the rate of harboring ARGs compared to females but there was no sex difference at 1 year. At one year, antibiotic exposure during initial hospitalization (*n* = 7) was associated with an overall lower risk of harboring ARGs compared to infants not given these antibiotics [relative risk: 0.41 (95% CI: 0.23–0.78)]. Infants exposed to penicillin-like intrapartum antibiotics had a 1.48 (95% CI: 1.10–2.00) times greater risk of harboring ARGs at 1 year compared to infants not exposed to intrapartum antibiotics. However, once we removed two 1-year samples that were considered outliers (RPKM of ARGs > 10,000), there was no association between penicillin exposure and relative abundance load of ARGs (Additional File 2: **Table S5**) but there was an association between resistome relative abundance and cesarean section [relative risk = 1.43 (95% CI: 1.12–1.84)]. To explore the interaction between intrapartum antibiotic exposure and delivery mode, we categorized the 210 infants into four groups: (1) vaginal delivery and no intrapartum antibiotic exposure (*n* = 89), (2) vaginal delivery and intrapartum antibiotic exposure (*n* = 63), (3) cesarean delivery and no intrapartum antibiotic exposure (*n* = 6), and (4) cesarean delivery and intrapartum antibiotic exposure (*n* = 52). With the inclusion of the two outliers, groups (2) and (4), which both had intrapartum antibiotic exposure, were both independently associated with harboring higher quantities of ARGs [relative risk = 1.30 (95% CI: 0.99–1.70) and relative risk = 1.54 (95% CI 1.16–2.07)] relative to group (1). Without inclusion of the outliers which both occupied group (2), only group (4) remained statistically significant [relative risk = 1.51 (95% CI: 1.16–1.96)]. Additional sensitivity analysis restricted to vaginal delivery suggested a similar trend; intrapartum antibiotics was not independently a risk factor for ARG load. Feeding mode was not statistically significant at either time point.

**Fig. 2:**
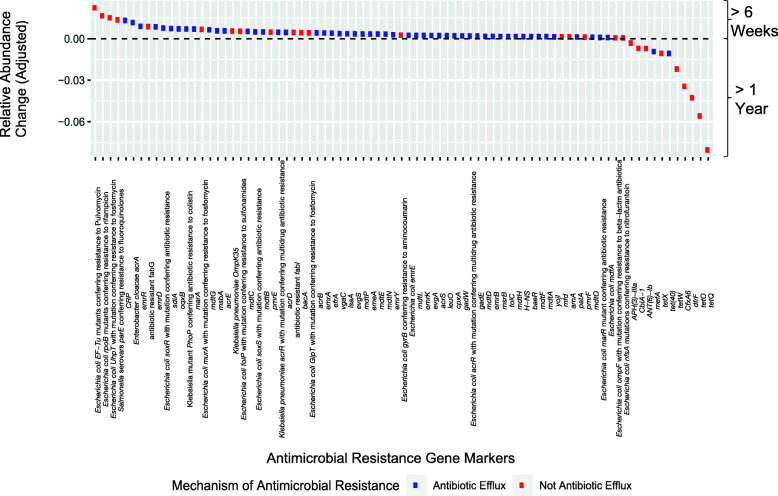
The relative abundance of antimicrobial resistance genes is different at 6 weeks and 1 year. MaAsLin2 was used to test if the compositional relative abundance of ARGs varied by postnatal age of the infant, delivery mode, feeding mode, gestational age at birth, infant sex, and antibiotic use during the infant’s initial hospitalization. Using a multiple hypothesis correction [Benjamini-Hochberg *q* < 0.01], 81 antimicrobial resistance genes were differentially abundant between the 6-week and 1-year time points. Antimicrobial resistance genes are colored by mechanism of antibiotic resistance (antibiotic efflux or not) with a greater proportion of genes that work through antibiotic efflux at 6 weeks (chi-square test *p* < 0.01)

After assessing overall measures of the resistome and determining differences by covariates and early-life factors, we hypothesized that these factors may be associated with the relative abundance of each unique ARG marker. We used MaAsLin2 [41] to test if the relative abundance of ARGs varied by covariates and early-life exposures (Additional File 2: **Table S6**). We found ARGs associated primarily with postnatal age of the infant. A statistically significant majority (chi-squared test *p* < 0.01) worked through the mechanism of antibiotic efflux (Fig. 2). The only early-life factor that was strongly associated with specific unique ARG markers was delivery mode. ARG markers associated with increased relative abundance in infants delivered via cesarean section were *E. cloacae acrA,* CRP (ARO: 3000518), and *sdiA* (ARO: 3000826) all of which encode resistance-nodulation-cell division antibiotic efflux pumps. Additionally, *Escherichia coli soxS* with mutation conferring antibiotic resistance (ARO: 3003511), *E. coli UhpT* with mutation conferring resistance to fosfomycin (ARO: 3003890), and *E. coli rpoB* mutants conferring resistance to rifampicin (ARO: 3003288), were identified and are linked to a variety of pathogenic species [35].

### Resistome composition is impacted by early-life factors primarily via *E. coli*

We hypothesized that certain species within the Proteobacteria phylum would be correlated with our overall resistome outcomes. The only species we determined to be strongly correlated (Spearman correlation > 0.5) with either outcome were *Escherichia unclassified* and *Escherichia coli*. Based on this initial result and previous research that has linked *E. coli* to ARG abundance [13, 19, 40], we decided to focus on this species.

**Fig. 3:**
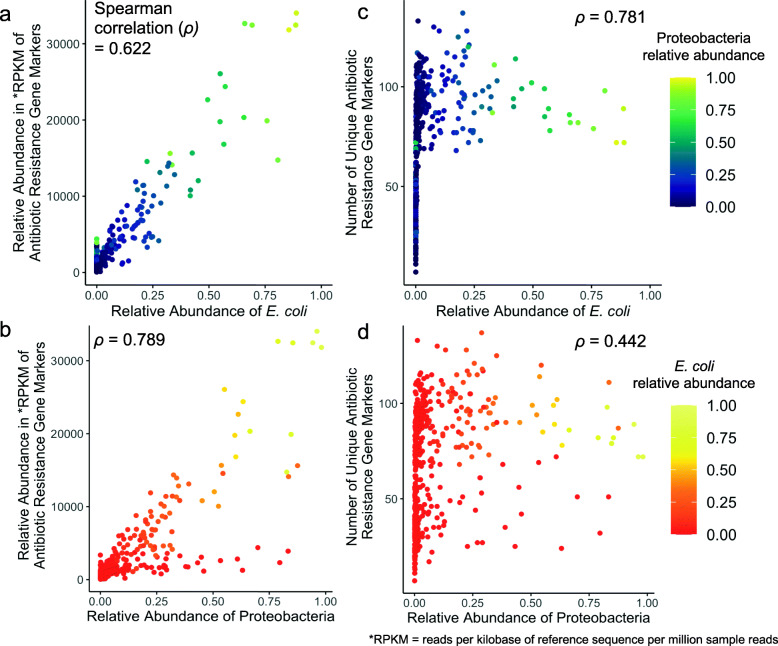
Scatterplots show the association between highly correlated taxa and overall resistome outcomes across 6-week and 1-year samples. Plots (**a**) and (**b**) depict the overall relative abundance of the resistome in RPKM versus the relative abundance of *E. coli* and Proteobacteria with high correlation. The number of unique antimicrobial resistance genes was most correlated with the relative abundance of *E. coli* (**c**) and less correlated with Proteobacteria relative abundance (**d**). **Fig.** **3a** and **c** are colored by the relative abundance of Proteobacteria and **Fig.** 3**b** and **d** are colored by the relative abundance of *E. coli*

To directly determine the association between *E. coli* and Proteobacteria with overall resistome outcomes, we plotted the relative abundance of each versus the resistome overall relative abundance (Fig. 3**a** and Fig. 3**b**) and number of unique ARGs (Fig. 3**c** and Fig. 3**d**) from all 420 samples. We found that the overall relative abundance of the resistome was correlated with Proteobacteria (78.9%) and *E. coli* (62.2%) relative abundance. Specifically for Fig. 3**b**, the samples with high Proteobacteria relative abundance and high ARG relative abundance were samples with high *E. coli* relative abundance. The relative abundance of *E. coli* had a 78.1% correlation with the number of unique ARGs, but only 44.2% correlation with Proteobacteria relative abundance. Unlike Fig. 3**a**, the association shown in Fig. 3**c** is not linear (i.e., no positive or negative association). Instead, we found a change point when the relative abundance of *E. coli* is 0.0038 (95% bootstrap CI: 0.0026, 0.0053). This indicates that *E. coli* relative abundance and the number of unique ARGs are positively associated (*p*-value < 0.001) when *E. coli* relative abundance is less than 0.0038. However, after *E. coli* relative abundance reaches this threshold (~ 0.4%) in the infant gut microbiome, it is not positively associated with the number of unique ARGs (*p*-value < 0.001). In other words, once the relative abundance of *E. coli* reaches 0.0038, a minimum number of ARGs is reached (43 unique ARGs) and there is no positive association between increasing relative abundance of *E. coli* and the number of unique ARGs.

**Fig. 4:**
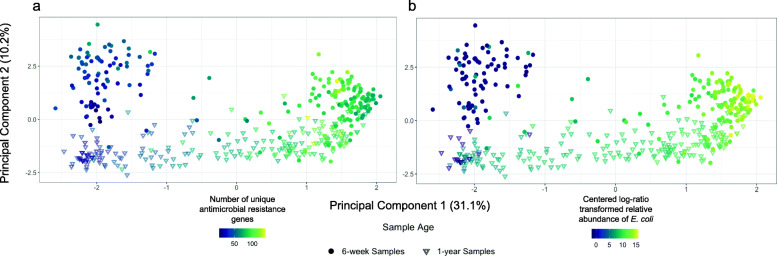
Infant gut sample resistomes are correlated with *E. coli* relative abundance. Principal component analysis (PCA) of centered log-ratio transformed relative abundance of infant gut resistomes colored by (**a**) the number of unique antimicrobial resistance genes and (**b**) centered log-ratio transformed *E. coli* relative abundance. Samples collected at approximately 6 weeks are represented as filled-in circles while samples collected at approximately 1 year are noted with triangles

Between sample (beta) resistome diversity using centered log-ratio (CLR) transformed ARG relative abundance and principal component analysis (PCA) was used to further understand which covariates or taxa were associated with the dispersion of samples’ resistome compositions. The PCA was described by two main principal components explaining 31.1 and 10.2% of the variation respectively. Upon visual inspection, we found that infant age was associated with principal component 2. In an attempt to understand the bimodal distribution of unique ARG markers (Additional File 1: **Fig. S1a**), we colored the PCA by the number of unique ARGs. The coloring aligned with the horizontal of principal component 1 sample separation (Fig. 4**a**). We were interested in assessing the underlying mechanisms for this dispersion including which factors led to variance in the number of unique ARG markers, so we considered the impact of *E. coli* relative abundance and early-life factors. Consistent with the results of the scatterplots associating *E. coli* relative abundance with other resistome outcomes (**Fig.** **3****a** and **c**), we found that *E. coli* relative abundance best described the dispersion patterns in the PCA (Fig. 4**b**). This aligns with our threshold regression that identified a strong association between the relative abundance of *E. coli* and the number of unique ARGs before the relative abundance of *E. coli* reached the change point of 0.0038. To statistically test whether *E. coli* composition described the dispersion of resistomes across samples, we conducted a PERMANOVA on the resistome composition adjusting for CLR-transformed relative abundance of *E. coli*, covariates, early-life factors, and individual variation. We found the relative abundance of *E. coli* to be associated with 24.7% of the variation of the relative abundance of the resistome. In comparison, each individual’s resistome described 35.3% of the variation. Additionally, sample age described 5.0% of the variation and feeding mode 2.4%, but no other factor described more than 1% of the variation (Additional File 2: **Table S7**).

**Fig. 5:**
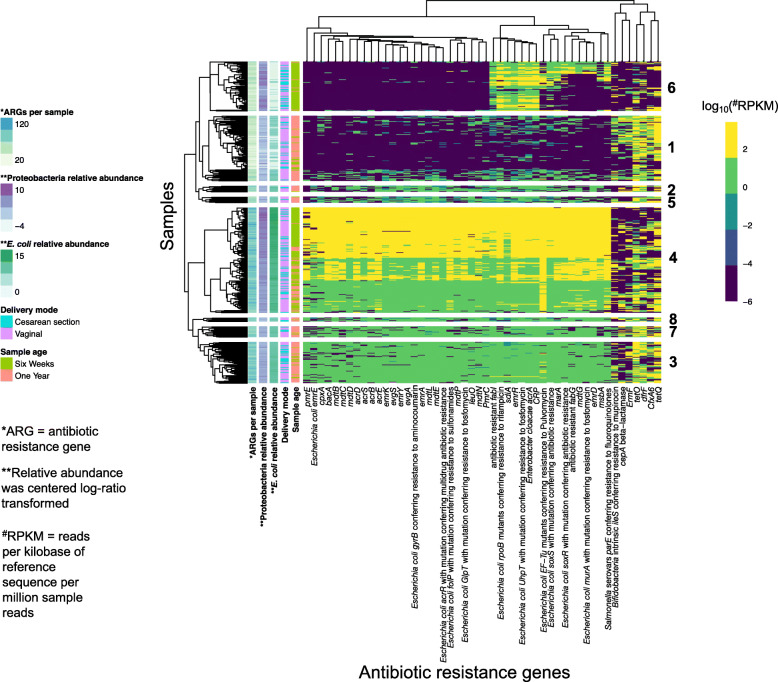
Heat map showing the 50 most abundant antimicrobial resistance genes (ARGs) by mean relative abundance. ARG (*x*-axis) relative abundances in RPKM have been log_10_-transformed and clustered by specific features of the samples (*y*-axis) including sample age, *E. coli* relative abundance, Proteobacteria relative abundance, delivery mode, and the number of unique ARGs per sample. ARGs are clustered by the Euclidean distance and samples are clustered using the Canberra distance. Hierarchical clustering of samples was used to determine 8 clusters as indicated by the numbers to the right of the heat map

To further explore how *E. coli* relative abundance may affect the relative abundance of ARGs, we plotted each sample against the 50 ARGs with the highest mean relative abundance across all samples using a heat map. Samples clustered mostly by the number of unique ARG markers and CLR-transformed *E. coli* abundance (Fig. 5). Of the 50 ARGs that had the greatest mean relative abundance, CARD listed *E. coli* as a potential species of origin in 36 of them (72%). Samples in cluster 4 had the highest relative abundance of *E. coli*, indicating a pattern of ARGs (from *pmrE* to *PmrC*) associated with *E. coli* relative abundance. We investigated the two clusters that encompassed the low ARG and *E. coli* relative abundance: (1) and (6). Cluster 1 (*n* = 95 samples) primarily consisted of 1-year samples (79/95 or 83%), and cluster 6 (*n* = 72 samples) primarily consisted of 6-week samples (67/72 or 93%), but, out of the 167 samples in the two clusters, only 48 samples were a paired 6-week sample from cluster 6 with a 1-year sample from cluster 1 indicating limited infant overlap. Interestingly, cluster 6 had a high relative abundance of Proteobacteria and ARGs including *CRP* and *sdiA* that were not in high abundance in Cluster 1. This indicates that, at least for Cluster 6, these ARGs may derive from a Proteobacteria other than *E. coli*. As the 7 ARGs on the far right of the heat map have distinct profiles from the other ARGs and are in low abundance in cluster 6 which had a high relative abundance of Proteobacteria, we hypothesize that the origins of these 7 ARGs including *tetO* and *tetQ* derive from a phylum other than Proteobacteria. Similar patterns were discernible when plotting the ARGs based on their prevalence (Additional File 1: **Fig. S3**). For instance, samples clustered primarily by *E. coli* relative abundance and the number of ARG markers per sample. Likewise, many ARGs across both heat maps clustered together including *tetQ*, *tetO*, and *dfrF* as well as *emrR* (ARO: 3000516), *CRP*, and *E. cloacae acrA*.

To test if microbiome or resistome composition progressed asymmetrically due to early-life exposures, we took advantage of the paired sample design and tested if CLR-transformed relative abundance of ARGs, number of unique ARGs, or CLR-transformed relative abundance of *E. coli* was differential between the two time points. Using adjusted linear regression models, we found that cesarean section delivered infants had on average 18.5 (4.6–32.4) additional unique ARG markers that increased from 6 weeks to 1 year compared to vaginally delivered infants (Additional File 1: **Fig. S4a**). Infants born by cesarean section also had on average 4.1 (95% CI: 1.7–6.4) units higher CLR-transformed relative abundance of *E. coli* compared to vaginally born infants (Additional File 1: **Fig. S4b**; Additional File 2: **Table S8**). This was not a result of differential sample collection ages between the 6-week and 1-year time points by delivery mode. These results suggest that cesarean delivery may be associated with differential acquisition of *E. coli* over time which may lead to increased relative abundance of ARGs.

Since we found *E. coli* to be strongly correlated with the number of ARG markers and early-life exposures, we hypothesized that early-life factors proliferate *E. coli* strains with unique genes. We used PanPhlan [42] to determine if the dominant strain of *E. coli* for each sample had different types of genes due to any early-life exposure. A PCA and heat map revealed that the gene presence/absence matrix separated into three groups predominantly based on the number of genes present and absent (Additional File 1: **Fig. S5a**). The first principal component of the strain analysis described 51.2% of the variation and was visually determined to be the total number of genes present and not related to any covariates (Additional File 1: **Fig. S5b**). A logistic regression analysis assessing sample age, feeding mode, mode of delivery and intrapartum antibiotic exposures, and number of reads (with gene presence/absence as the outcome) identified one gene to be statistically significantly associated with increased presence in 1-year samples after a multiple hypothesis correction (*q* < 0.1). A BLAST search of the gene using the Kyoto Encyclopedia of Genes and Genomes (KEGG) revealed it was a 23S ribosomal RNA gene. Mutations within 23S ribosomal RNA are associated with different types of antibiotic resistance (ARO: 3000336). As this gene was only associated with sample age, this finding suggests that no individual ARG within the *E. coli* pangenome was independently associated with early-life exposures beyond postnatal age in our dataset.

Lastly, we assessed if mobile genetic elements (MGEs) were associated with infant characteristics and *E. coli* relative abundance. Using results from HUMAnN2, we identified three MGEs related to transposition (GO:0004803 [molecular function] transposase activity; GO:0006313: [biological process (BP)] transposition, DNA-mediated; and GO:0032196 [BP] transposition) and one related to plasmid maintenance (GO: 0006276 [BP]). We tested each MGE versus infant characteristics and early-life factors using MaAsLin2. We found that all of the MGEs were statistically significantly associated with sample age with transposase activity and DNA-mediated transposition at a greater relative abundance at 1 year while transposition and plasmid maintenance were at a greater relative abundance at 6 weeks (Additional File 2: **Table S9**). For plasmid maintenance in particular, *E. coli* was an important contributor to the MGE’s relative abundance (Additional File 1: **Fig. S6**).

## Discussion

In this prospective cohort study of paired 6-week and 1-year stool samples from 210 infants, we characterized the developing infant gut resistome and determined which factors affect its composition. Results from this resistome epidemiology study align with previous studies; postnatal age of the infant and taxonomic composition drive both individual ARGs and overall resistome composition but other factors play a role either directly or indirectly including delivery mode, feeding method, and sex. Overall, our results provide insight into the acquisition and development of ARGs in a general population cohort of infants.

### The infant gut resistome changes primarily in relation to the microbiome

The infant gut resistome samples profiled at 6 weeks were different than those profiled from the same infants at 1 one year of age, which we primarily attribute to shifts in microbial composition. We found a higher relative abundance of ARGs at 6 weeks than 1 year. This finding agrees with other studies analyzing the resistome that have found greater relative abundances of ARGs in younger infants compared to young children or adults [11, 15, 18, 19]. Additionally, although we did not identify a difference in the observed number of unique ARGs across the two time points, the types of ARGs varied. These trends occurred in parallel to the taxonomic composition development within the infant gut. Specifically, the 6-week samples had a high relative abundance of Proteobacteria compared with 1 year samples. In our study, the relative abundance of both *E. coli* and Proteobacteria were highly correlated with the composition of the resistome.

Our findings agree with other studies that have identified Proteobacteria [13, 15, 19, 39, 40] and specifically *E. coli* [13, 19, 40] as significant sources of ARGs. While we cannot directly assess causation, we found that specific ARGs, clusters of ARGs, the overall relative abundance of ARGs, and the number of unique ARGs were all associated with *E. coli* or Proteobacteria. While it could be argued that these results are a consequence of identified ARGs, it is worth noting that studies across different species and ecosystems using qPCR or assembly-based methods have also identified that Proteobacteria harbor a proportionately high level of ARGs [43, 44]. Additionally, although *E. coli* are commonly found in low abundance within the human gut and are recognized as early colonizers of the infant gut, microbial dysbiosis can lead to Proteobacteria and *E. coli* blooms presenting increased opportunities for horizontal gene transfer of ARGs [45, 46]. As *E. coli* contain a high diversity of plasmids and high potential for horizontal gene transfer [46], this is a particular concern. Two recent papers [14, 19] did not directly identify an association between any specific MGE and *E. coli* in the infant gut, but do provide additional context that support this study’s findings. Of particular interest, one study found that *E. coli* relative abundance was associated with an increased ARG relative abundance and that plasmids and other MGEs were at a higher relative abundance in infant guts at 1 month as opposed to 6 months [19]. Both studies also found MGEs to be at a greater abundance in infants as opposed to mothers [14, 19]. This notion that *E. coli* is important to the resistome and mobilome is also supported by the results of our MGE analysis as MGE relative abundance was associated with *E. coli*-specific contributions especially regarding plasmid maintenance. Future studies with additional timepoints before 1 year could evaluate in detail the dynamics of *E. coli* abundance in the gut and how they relate to the development of the gut resistome over time. Findings from these studies could provide insight on *E. coli* susceptibility patterns to available antibiotics in early life and could be used to evaluate appropriate empiric regimens to early-life infections and bacteremia. For instance, as both Group B *Streptococcus* and *E. coli* are leading causes of neonatal sepsis [47, 48], it is becoming increasingly important to assess how intrapartum antibiotic prophylaxis for preventing Group B *Streptococcus* infections may affect ARGs in *E. coli* and lead to antibiotic resistant *E. coli* infections.

### Delivery mode and not intrapartum antibiotic exposure contributes to resistome alterations

Intrapartum antibiotic exposure and delivery mode have previously been rarely explored in full term infant gut resistome epidemiology. Only one study [19] has specifically assessed intrapartum antibiotic exposure on ARGs in infant stool. The study included 16 vaginally delivered, full term (at least 37 weeks’ gestation) infants assessed at approximately 1 and 6 months. They found that intrapartum antibiotic exposure did not impact overall resistome composition although some ARGs [including *dfrE*, *efrA*, and *lsa(A)*] profiled were at a higher abundance among infants in the exposed group. We did not find evidence that any ARG was at a higher relative abundance after adjustment for other covariates and after accounting for possible false positives through multiple hypothesis correction. Differences between studies may be a result of varying sample age measurement; differential measurement of taxa and genes; variation in the dose, duration, and type of intrapartum antibiotic administered; or differential sensitivities to intrapartum antibiotics that vary by study population.

Delivery mode has also been associated with differential resistome load. One study [15] found that infants that were vaginally delivered had a lower proportion of ARGs (as a fraction of the overall number of genes) in their samples compared to cesarean delivered infants in newborn, 4-month, and 12-month stool samples. We found that 6 ARGs were at an increased relative abundance in infants born via cesarean delivery as compared to those delivered vaginally. These genes have been identified in sequences of *Salmonella enterica, E. coli,* and pathogens often associated with nosocomial transmission including some *Enterobacter* spp. and *Klebsiella pneumoniae* [35]. All of these ARGs also clustered together in Fig. 5. Of particular interest were the samples in cluster 6. This cluster of mainly 6-week samples had low *E. coli* relative abundance but high levels of Proteobacteria relative abundance. Based on the ARG and taxonomic patterns outlined in this group, it seems likely that these 6 ARGs, at least for infants in cluster 6, mainly derived from Proteobacteria other than *E. coli*. Additionally, this cluster had low ARG relative abundances for 7 ARGs that clustered together which we hypothesize came from non-Proteobacteria. As some of these 7 ARGs could have derived from *Bacteroides* and a significant portion of infants born by cesarean delivery have been shown to have a distinctly lower relative abundance of *Bacteroides* [12, 15, 20, 23], we hypothesize that the ARG clustering for infants in cluster 6 might be representative of a low-*Bacteroides* resistome. This hypothesis aligned with the taxonomic data; the mean relative abundance of *Bacteroides* was statistically significantly different (*t*-test *p*-value < 0.001) when comparing Cluster 6 samples to samples in other clusters. The mean relative abundance of *Bacteroides* in Cluster 6 was 6.5% versus 12.4% in the other clusters. Although there is variation in microbial succession regardless of delivery mode, cesarean section as shown in this study and others [12, 15, 20] can alter the timeline of taxonomic development within the infant gut. Thus, our conclusion is that cesarean section likely impacts resistome composition directly and indirectly via alterations to the succession of microbes in the infant gut.

Studies often have not been powered to tease apart intrapartum antibiotic exposure from other exposures that occur concomitantly at delivery including gestational age and delivery mode. This has contributed to inconsistent results regarding the independent impacts of intrapartum antibiotic exposure. Although our analysis of overall resistome load at 1 year (*n* = 210) found that intrapartum antibiotic exposure to penicillin was associated with an increased risk of harboring ARGs, upon sensitivity analyses to remove 2 infants with RPKM of ARGs greater than or equal to 10,000 at 1 year, this result no longer persisted. Further analyses reclassifying intrapartum antibiotic exposure and delivery mode into four groups suggested only the combination of cesarean delivery and intrapartum antibiotic exposure to be associated with an increased rate of harboring ARGs. While it is possible a positive association between intrapartum antibiotic exposure and the overall relative abundance of ARGs may exist only in infants that are born by cesarean section (i.e., cesarean section is an effect modifier), we hypothesize cesarean section to be the more important factor driving the increased relative abundance of ARGs for several reasons. First, it is worthwhile to note that the two 1-year samples that were removed had a high relative abundance of *E. coli* (21 and 41% respectively) compared to the average among 1 year samples (1.3% not including the outliers)*.* This may be suggestive of an *E. coli* bloom prior to or during sample collection for these two infants. While we cannot assess if the association between the high *E. coli* relative abundance in these two samples is related to intrapartum antibiotic exposure, we hypothesize that sudden increases to *E. coli* relative abundance at 1 year are likely not directly due to at-birth exposures but more likely to be a result of a short-term perturbation. Second, across multiple analyses, we identified positive associations between cesarean delivery and the resistome, but never with intrapartum antibiotics regardless of the antibiotic type. Lastly, in sensitivity analyses limited to infants who were delivered vaginally or by cesarean section, we found no association between intrapartum antibiotic exposure and overall relative abundance of ARGs. Additional studies that have more infants that were born via cesarean delivery but not exposed to intrapartum antibiotics (e.g., in cohorts such as the Microbiome Utrecht Infant Study where mothers were not given antibiotics until after the umbilical cord was clamped [24]) could be used to validate this conclusion. Since there was only a small number (*n* = 6) of infants who were delivered by cesarean section but did not receive intrapartum antibiotics in this study, this likely precluded our ability to statistically significantly detect the independent exposure of cesarean section. The higher ARG relative abundance in cesarean delivered infants could be due to the environment of the operating room, preferential colonization from skin as opposed to vaginal microbes, differences in mothers or infants who are born by cesarean delivery, or potentially a synergistic interaction between intrapartum antibiotics and cesarean delivery.

### Other covariates and early-life factors may alter the trajectory of the microbiome and indirectly impact the resistome

Feeding method has been considered one of the most important factors in microbial and metabolic development [30, 33], but we found little evidence to suggest its impact directly on the resistome. A previous study in preterm infants [21] identified class D beta-lactamase genes to be enriched in formula fed infants which were most frequently identified in *Clostridium difficile*. We did not find class D beta-lactamase genes in infants who were formula fed. The remainder of our results suggest feeding method acts on the resistome via increasing taxonomic diversity, which our group has previously shown is greater at 1 year compared to 6 weeks within the NHBCS infant population [26]. This increased taxonomic diversity could also be correlated with the introduction of formula or solid foods. For instance, compared to infants that were exclusively breastfed, infants that were given any formula at 6 weeks had an increased risk for developing unique ARGs but a lower risk at 1 year. This finding suggests that introduction of taxonomic diversity during unstable periods in development can lead to more instability, but in more stable infant gut communities, taxonomic diversity provides colonization resistance [49].

Surprisingly, we identified that male sex was associated with a greater overall relative abundance of ARGs at 6 weeks, but with a decreased risk of unique ARGs at 1 year. A recent study [14] found that among 40 infants that were 6 months old, males had a higher richness of ARGs (defined as the total prevalence of ARGs in each sample) but found no difference in the summed relative abundance. Another study [50] assessed sex differences in the gut resistome of adults. They found that females had a greater mean prevalence of ARGs than males and attributed this primarily to differences in antibiotic prescribing practices by sex. Sex differences in antibiotic prescription rates have not been fully teased apart [51] but, for older children and adults, some of the differences are likely due to distinct urinary tract infection rates or sex-based differences in care utilization [51, 52]. As these factors may not be as applicable for infants, antibiotic prescription rate differences may not explain the differential resistome composition. Instead, a combination of microbial, hormonal, or developmental differences may explain variation between infant male and female resistomes.

### Strengths and limitations

While our study had many strengths including our large sample size, paired sample design to reduce individual-level variation, and prospective data collection framework, our study does have some limitations. The NHBCS includes primarily White women and their infants from New Hampshire and Vermont in the United States. Thus, our results may not be generalizable to other communities with different ethnic [53] or geographic compositions [31]. Most variables were collected via standardized medical record review including age, sex, delivery mode, and antibiotic exposures reducing multiple possible biases. However, for reported covariates such as feeding mode within the first year, recall and misclassification bias was a concern. To mitigate this, we temporally assessed infant breast feeding exposures beyond the marked interval time points to improve the accuracy of the feeding mode classification (see Methods). Additionally, we conducted a duplicate analysis of one 6-week sample from sequencing to analysis. The duplicate samples were highly correlated across taxonomic and resistome metrics. We also did not include antibiotic exposures throughout the first year of life due to the variety and timing of the exposures, but recognize future studies that primarily focus on antibiotics over the first year of life should be conducted. Likewise, we acknowledge that there are inherent limitations to the reference databases, many of which are updated frequently, and tools we used to profile genes and taxa. To alleviate this concern, we used previously curated and highly used sources including a precompiled set of ARG markers from 2017. Thus, we believe any misclassification would be non-differential by infant characteristic. Specific to ARGs, there is much debate regarding the classification of ARGs and their actualized significance to differential health outcomes with some ARGs purely indicating the presence of a particular species [54]. Regardless, these genes can functionally confer resistance to the associated organism and is the predominant reason why we did not remove any possible ARGs from analysis. Future papers could work to tease apart which genes functionally contribute to antimicrobial resistant infections within and between hosts or use metatranscriptomics to further investigate the expression and utilization of these ARGs.

## Conclusion

We identified that the infant gut resistome of 210 infants from the general population sampled at two time points in the NHBCS varies by early-life exposures but predominantly tracks with taxonomic succession. Proteobacteria and specifically *E. coli* were correlated with the overall relative abundance of ARGs and the number of unique ARGs. Early-life factors and covariates were associated with the infant gut resistome. Cesarean delivery most associated with differential resistome acquisition and development. As one of the largest infant gut resistome epidemiology studies to date, this work provides a baseline level of resistome development in a general population of infants. Future studies should consider how other early-life factors and perturbations throughout development may impact the infant gut resistome to offer insights on measures to reduce antibiotic resistance early in life.

## Methods

### Study population and design

The NHBCS is an ongoing prospective cohort study of over 2000 pregnant women and their offspring recruited from prenatal clinics in New Hampshire, USA. Pregnant women ages 18–45 are recruited from prenatal clinics beginning at approximately 24–28 weeks of gestation and all women must use private well water as discussed previously [55]. Fecal samples were collected prospectively for infants at approximately 6 weeks and 1 year. Institutional review board approval was obtained at Dartmouth with yearly renewal. Written informed consent for participation was received from parents for themselves and their children.

### Covariate data collection

Extensive covariate data on lifestyle, medical history, and environmental exposures was collected in the NHBCS from participants through medical records, postpartum questionnaires, and telephone interviews. Delivery mode (vaginal or cesarean), feeding mode (exclusively breast fed, exclusively formula fed, or combination of breast and formula fed), prenatal maternal antibiotic usage (yes or no), gestational age (in days), birth weight (in grams), maternal Group B *Streptococcus* status (yes or no), infant sex (male or female), and other demographic characteristics were collected from telephone questionnaires at 4, 8, and 12 months following birth and delivery medical records. Age of breast milk samples is also used to determine feeding mode and additional information about the classification of breastfeeding exposure. Additional information about feeding method prior to infant stool sample collection can be found in Additional File 1: **Supplementary Methods**. Infant antibiotic exposures were only classified if oral, injected, or intravenous antibiotic exposure was indicated during the initial hospitalization. Intrapartum antibiotic exposures administered during labor and delivery were extracted from maternal medical delivery records and were assessed both as a yes/no variable and explored by class of intrapartum antibiotic received. Subjects were grouped according to intrapartum antibiotic exposures using the following categories: no antibiotics; penicillin-like antibiotics only (amoxicillin, penicillin); cephalosporins only (cefazolin, cephalexin); multi-drug classes (two or more antibiotics characterized as penicillin, cephalosporin, vancomycin, clindamycin, and/or gentamicin); or “other” antibiotics such as aminoglycosides, glycopeptides, or lincomycin.

### Metagenomics collection and processing pipeline

Infant stool samples were collected at 6-weeks and 12-months maternal postpartum. Stool samples were provided in diapers and stored by guardians in a home freezer (− 20 °C) until they were able to return them to the study site. Stool was thawed at 4 °C and aliquoted (range 350–850 mg) into 3 ml RNAlater in cryotubes and homogenized before storing at − 80 °C. RNAlater stool samples were thawed and DNA was extracted using the Zymo Fecal DNA extraction kit (Cat# D6010, Zymo Research, Irvine, CA), according to the manufacturer’s instructions. For each sample extraction, 400ul RNAlater stool slurry (50–100 mg of stool) was used to isolate DNA. Extractions were performed in batches of multiple samples and included a composite RNAlater stool positive control and a RNAlater negative control. Lysis of stool slurry was performed using 750ul Lysis Buffer in ZR BashingBead™ Lysis Tubes (0.5 mm beads), mixed and then shaken on a Disruptor Genie for 6 min. Eluted DNA was quantified on a Qubit™ fluorometer using the Qubit™ dsDNA BR Assay. Average coefficient of variation of DNA yields (ng/ul) for composite RNAlater stool positive controls was 28%. No DNA was ever detectable in negative control elutions. Concentrations of DNA samples used for metagenomic gene sequencing ranged from 1 ng/ul to 25 ng/ul.

Metagenomic sequencing libraries were prepared at the Marine Biological Laboratory (MBL) in Woods Hole, MA using established methods. These libraries were constructed using Nugen’s Ovation Ultralow V2 protocol. Using a Covaris S220 focused ultrasonicator, DNA samples were sheared to a mean insert size of 400 base pairs.

Some metagenomic samples were processed as paired-end reads (*n* = 219; 52.1%) and others as single-end reads (*n* = 201; 47.9%). Generally, sequencing batches of 12 were run for paired-end reads and batches of 16 were run for single-end reads. Paired-end DNA reads were merged into one FASTQ file and all samples were trimmed with KneadData and Trimmomatic with default settings [56] for quality control. Mean (SD) reads after this quality control for single-end reads was 22.3 million (9.6 million) and was 58.9 million (20.8 million) for paired-end reads.

Taxonomic analysis was conducted using both MetaPhlAn2 [38] and PanPhlAn version 1.2.2.5 (10 May 2018) [42]. MetaPhlAn2 [38], an output of the HUMAnN2 [57] version 0.11.2 pipeline, was used to analyze relative abundance of taxa to the species level. MetaPhlAn2 characterizes microbial clades through the use of clade-specific markers. PanPhlAn was used to conduct a strain-level analysis of *E. coli*. A precompiled pangenome database of *E.coli* from 2016 [42] was utilized for this analysis. Using PanPhlAn, each sample was mapped to the pangenome to assess presence and absence of genes corresponding to the dominant strain of *E. coli* in each sample.

ARG markers were quantified using ShortBRED version 0.9.5 [34]. ShortBRED works in two steps. First, it creates a database of antimicrobial resistance gene markers and then uses this set of makers to identify antibiotic resistance genes in samples. A precompiled list of markers [58] known to confer bacterial antibiotic resistance from CARD version 1.1.8 [35] was used and the relative abundance of ARG markers in our samples were classified using the “shortbred_quantify” script with default parameters. Outputs from ShortBRED are normalized for average read length, marker length, and sequencing depth and are represented in RPKM. Annotations for the ARGs were adapted from [50], which also used ARG markers from ShortBRED. The current version of the CARD database (3.1.0) profiles 88 potential pathogens to assess if the ARG exists within species’ resistomes using an antimicrobial resistance detection model [35]. While this taxonomic origin information via CARD is worthwhile to note, these ARGs could be derived from other species not profiled directly by CARD. Additionally, some ARGs contain the name of an organism, but CARD frequently identifies multiple potentially pathogenic species’ resistomes that the ARG may be present in. For instance, CARD says that *E. cloacae* has a perfect resistome match to *E. cloacae acrA*, but the gene has also been found in other *Enterobacter* species and *Klebsiella pneumoniae’s* resistomes with sequence variants.

MGEs were identified using HUMANn2 [57] with default settings. Briefly, HUMAnN2 uses a ‘tiered search’ approach to first identify known species using reference markers, map these reads to the pangenome of the species, and finally conduct a translated search on all reads that were not classified by known species giving gene family in reads per kilobase. Output gene family files were regrouped by gene ontology (GO), normalized to relative abundance to account for sequencing depth, merged into one file, and then renamed. This was completed using the “humann2_regroup_table”, “humann2_renorm_table”, “humann2_join_tables”, and “humann2_rename_tables” utility scripts available via bioBakery [59].

### Baseline metrics of the resistome

The prevalence, mean, and median values for all ARG markers were assessed overall and stratified by 6-week and 1 year samples. Additionally, all values were assessed in comparison to all samples and to only samples that had the gene present. ARGs were considered present if they were present in at least one sample.

### Covariate selection

Early-life exposures and variables that we hypothesized to be associated with differential resistome composition were chosen based on previous research demonstrating their impact on the resistome and due to their specificity. For our models we used the following covariates: postnatal age of the infant (sampled at approximately 6 weeks or 1 year), gestational age (in weeks), sex (male or female), delivery mode (vaginal or cesarean section), intrapartum antibiotic exposure (yes or no), feeding mode (exclusively breastfed, exclusively formula fed, or mixed fed at the time of sample collection), and infant antibiotic exposure before leaving the hospital (yes or no). Sensitivity analyses assessed sample age in days as a linear variable with the infant as a random effect, intrapartum antibiotic exposures grouped by class of antibiotic prescribed, never versus ever formula fed, and possible joint interactions between intrapartum antibiotic exposure (yes or no) and delivery mode (vaginal or cesarean delivery). Inter-individual differences were analyzed as it often accounts for the largest amount of variation in microbiome studies [20, 30, 60]. Geographic location has also been associated with differential resistome composition [39], but is controlled primarily through restricting to NHBCS infants.

### Statistical analysis

An overview of the methods and statistical tools used is provided in Additional File 1:**Fig. S7**. The impacts of covariates on the resistome were assessed primarily using negative binomial regression through quantifying two outcomes: i) the relative abundance of all ARG markers and ii) the presence of unique ARG markers. Additionally, as microbes are hypothesized to be on the causal pathway between many early-life exposures and the resistome, we tested how taxa are correlated with covariates, overall relative abundance of ARGs, and number of unique of ARGs. The outcomes were analyzed across all our samples controlling for sample age (6-week or 1-year) and stratified by the age of sample collection (i.e., cross-sectionally). Negative binomial regression was selected over regression models using the normal or Poisson distribution to avoid overdispersion [19] and because the coefficient can be exponentiated to estimate relative risk. Thus, the interpretation of the negative binomial regression results for assessing the overall number of unique ARGs would be: in comparison to the unexposed group, the exposed group had X times the risk of harboring unique ARGs. Relative risks can be considered statistically significant if they do not include 1, but, if they do cross 1, may be considered meaningfully significant depending on the width of the confidence interval. To assess relative abundance of ARG markers and alpha diversity of species together, Shannon alpha diversity metrics were extracted for species and used as an additional exposure with overall ARG marker relative abundance and number of ARG markers observed as outcomes in sensitivity analyses. To assess the direct correlation between microbial relative abundance and the two overall resistome outcomes, we assessed the Spearman correlation between each species and phyla relative abundance against each outcome. A threshold effect was hypothesized between the relative abundance of *E. coli* and the number of unique ARGs based on Fig. 3**c**. We used the R package “chngpt” [61] and tested this hypothesis using a segmented threshold regression model with the null hypothesis that there was no threshold effect that could describe the association between *E. coli* relative abundance and the number of unique ARGs.

Phyloseq [62] objects were created to measure diversity metrics and to make compositional plots for ARGs, taxa, and functional analyses. Alpha (within) sample diversity metrics were calculated using the Shannon and Simpson diversity metrics. For beta (between) sample diversity, genes in RPKM were transformed into compositional data. Using the “microbiome” package [63], a pseudo count of the minimum value divided by two was used in place of any 0s and then the data was CLR-transformed [64]. PCA plots using Euclidean distances were created to visualize results by different covariates. A PERMANOVA was created using the *adonis2* function in “vegan” [65] to evaluate between sample diversity.

To further explore similarities and differences in groups with adjustments for all other covariates, MaAsLin2 [41] was used. MaAsLin2 uses a feature reduction technique involving additive boosting of generalized linear models to choose covariates that are most associated with the outcome of interest. Compositional abundance data for each ARG was associated with covariates in MaAsLin2. Deviation from the default parameters included the use of a CLR normalization approach, no standardization of continuous variables, no transformation, and only associations with Benjamini-Hochberg multiple hypothesis correction (*q*-value) less than 0.01 were considered statistically significant.

As we were interested in understanding how early-life exposures and taxa would impact individual and clusters of ARGs, we visualized associations with heat maps using the “pheatmap” [66] package in R. We first assessed the association between the 50 ARGs with the highest mean relative abundance across all samples. Any ARG with a relative abundance of 0 was given a pseudo-value of 0.000001, and then the ARG relative abundances in RPKM were log_10_-transformed. ARGs were clustered using the Euclidean distance and samples were clustered using the Canberra distance based on previously used clustering methods [34]. Hierarchical clustering was used to delineate between groups of samples and 8 clusters were selected in order to visualize samples clustering based on low *E. coli* relative abundance (clusters numbered 1 and 6). A second heat map of these associations was created that displayed ARGs present in at least 60% of samples. ARGs were considered present if they had a RPKM relative abundance of at least 0.01. Both samples and ARGs were clustered using the Euclidean distance.

To understand whether *E. coli* strains varied by any covariates in our analysis, we used a variety of visual and statistical tools. Multidimensional scaling using the jaccard distance on the presence/absence matrix of genes with a prevalence of at least 10% was used to visualize sample similarities and differences by covariates. A heat map including only genes that had between 20 and 80% prevalence was used to cluster samples. Logistic regression with a Benjamini-Hochberg correction (*q* < 0.1) was used to assess if any genes were differentially abundant by sample age, feeding mode, a combination of delivery mode and intrapartum antibiotic exposure, or total number of reads. A BLAST search of statistically significant genes using KEGG was conducted to reveal differentially prevalent genes.

The goals of the MGE analysis were twofold as we wanted to assess if MGE relative abundance was associated with (1) any covariate or (2) the species-specific contribution of *E. coli*. Using a custom R script, we collapsed all taxonomic information in the resulting gene families file outputted by HUMANn2 to calculate the relative abundance of each GO term for each infant sample (i.e., the summed gene family relative abundance for each sample equaled 1). Using a CLR-normalization, MaAsLin2 was used to assess which gene families were statistically associated with metadata including sample age, feeding method (ever or never formula fed), intrapartum antibiotic exposure, delivery mode, gestational age, infant sex, and antibiotic use during the initial hospitalization. Statistically significant results (*q*-value < 0.25) were queried for MGEs. Specifically, the terms “integrase”, “integron”, transpos”, or plasmid” were used for the query as they previously have been used to identify MGE elements from HUMAnN2 results [67]. Since we found these MGEs to only be associated with sample age, we assessed the species specific contribution to each MGE broken down by sample age using the “humann2_barplot” script from HUMAnN2. Bar plots were sorted by the sample sum coefficient of the GO term and grouped by sample age.

### Quality control

Number of reads was not added as a covariate in any models where a relative abundance was calculated as all techniques normalized for sample depth (number of reads). However, our assessment of unique ARG markers and genes present in *E. coli* included log_10_-transformed number of reads as these presence/absence analyses were impacted by sample depth. Sequencing type and batch effects were assessed through a PCA of the resistome. No evidence of differential effects to the resistome were identified in either (Additional File 1: **Fig. S8a** and **Fig. S8b**) so sequencing type and batch effect were not considered as a covariate in regression models. A secondary quality control measure was assessed by rerunning a 6-week, single-end sample through the sequencing and downstream analysis pipeline. Upon re-analysis, ShortBRED and MetaPhlAn2 results were nearly identical (Additional File 1: **Fig. S8c** and **Fig. S8d**).

## Supplementary Information

**Additional file 1 Fig. S1:** Relative abundance and distribution of number of unique antimicrobial resistance gene markers by sample age. **Fig. S2:** Longitudinal change in relative abundance of ARG markers colored by mechanism of antibiotic resistance (antibiotic efflux or not). **Fig. S3:** Heat map showing the most prevalent antibiotic resistance genes (present in at least 60% of samples) by sample. **Fig. S4:** Differential unique antimicrobial resistance genes and *E. coli* relative abundance between paired samples by delivery mode. **Fig. S5:** *E. coli* strains do not have differential genes based on early-life exposures. **Fig. S6:** Four gene families related to mobile genetic elements are associated with sample age and specific taxa. **Fig. S7:** Overview of Analytic Steps and Main Statistical Methods. **Fig. S8:** Quality control to assess the validity of the sequencing and downstream analysis tools. **Supplementary Methods:** Infant Feeding Mode up to the Time of Stool Sample Collection.

**Additional file 2 Table S1:** Baseline Characteristics by Single vs. Paired-end Reads. **Table S2:** Prevalence, Mean, and Median of ARGs by Sample Age. **Table S3:** Negative Binomial Regression of Unique ARG Markers. **Table S4:** Negative Binomial Regression of Relative Abundance of ARGs. **Table S5:** Analysis of 2 Infants with Overall ARG Relative Abundance > 10,000 at 1 Year. **Table S6:** MaAsLin2 Analysis of Antimicrobial Resistance Genes Adjusted for Covariates and Early-life Exposures. **Table S7:** PERMANOVA of the Resistome. **Table S8:** Linear Models to Assess Difference between 1-Year and 6-Week Samples by Covariates. **Table S9:** MaAsLin2 Analysis of HUMAnN2 Gene Families Adjusted for Covariates and Early-life Exposures.

## Data Availability

The whole metagenomic shotgun sequencing samples are available through the National Center for Biotechnology Information (NCBI) Sequence Read Archive: https://www.ncbi.nlm.nih.gov/sra (Accession number: PRJNA296814).

## Abbreviations

ARG: antimicrobial resistance gene
ARO: Antibiotic Resistance Ontology
CARD: Comprehensive Antibiotic Resistance Database
CLR: centered log-ratio
CI: confidence interval
NHBCS: New Hampshire Birth Cohort Study
MGE: mobile genetic elements
PCA: Principal components analysis
RPKM: reads per kilobase of reference sequence per million sample reads

## References

[1] World Health Organization. Antimicrobial Resistance Global Report on Surveillance. 2014.

[2] Perry JA, Westman EL, Wright GD. The antibiotic resistome: What’s new? Curr Opin Microbiol [Internet] 2014;21:45–50. Available from: 10.1016/j.mib.2014.09.002 10.1016/j.mib.2014.09.002 25280222

[3] Wright GD. The antibiotic resistome: the nexus of chemical and genetic diversity. Nat rev Microbiol [internet]. 2007;5:175–86. Available from: http://www.ncbi.nlm.nih.gov/pubmed/17277795. 10.1038/nrmicro1614 17277795

[4] Hernando-Amado S, Coque TM, Baquero F, Martínez JL. Defining and combating antibiotic resistance from one health and Global Health perspectives. Nat Microbiol [Internet]. 2019;4:1432–1442. Available from: 10.1038/s41564-019-0503-9 10.1038/s41564-019-0503-9 31439928

[5] Pal C, Bengtsson-Palme J, Kristiansson E, Larsson DGJ. The structure and diversity of human, animal and environmental resistomes. Microbiome [Internet] 2016;4:1–15. Available from: 10.1186/s40168-016-0199-5 10.1186/s40168-016-0199-5 PMC5055678 27717408

[6] Pehrsson EC, Tsukayama P, Patel S, Mejía-Bautista M, Sosa-Soto G, Navarrete KM, et al. Interconnected microbiomes and resistomes in low-income human habitats. Nature [Internet]. 2016;533:212–6. Available from: 10.1038/nature17672 10.1038/nature17672 PMC4869995 27172044

[7] Salyers AA, Gupta A, Wang Y. Human intestinal bacteria as reservoirs for antibiotic resistance genes. Trends Microbiol [Internet]. 2004;12:412–6. Availablefrom: 10.1016/j.tim.2004.07.004 10.1016/j.tim.2004.07.004 15337162

[8] Moore AM, Patel S, Forsberg KJ, Wang B, Bentley G, Razia Y, et al. Pediatric fecal microbiota harbor diverse and novel antibiotic resistance genes. PLoS One [Internet]. 2013;8:e78822. Available from: 10.1371/journal.pone.0078822 10.1371/journal.pone.0078822 PMC3827270 24236055

[9] Hicks LA, Bartoces MG, Roberts RM, Suda KJ, Hunkler RJ, Taylor TH, et al. US outpatient antibiotic prescribing variation according to geography, patient population, and provider specialty in 2011. Clin infect dis [internet]. 2015;60:1308–16. Available from: 10.1093/cid/civ076. 10.1093/cid/civ076 25747410

[10] Lee GC, Reveles KR, Attridge RT, Lawson KA, Mansi IA, Lewis JS, et al. Outpatient antibiotic prescribing in the United States: 2000 to 2010. BMC Med [Internet]. 2014;12:1–8. Available from: 10.1186/1741-7015-12-96 10.1186/1741-7015-12-96 PMC4066694 24916809

[11] Gibson MK, Wang B, Ahmadi S, Burnham CD, Tarr PI, Warner BB, et al. Developmental dynamics of the preterm infant gut microbiota and antibiotic resistome. Nat Microbiol [Internet] 2016;1:1–25. Available from: 10.1038/nmicrobiol.2016.24 10.1038/nmicrobiol.2016.24 PMC5031140 27572443

[12] Yassour M, Vatanen T, Siljander H, Hämäläinen A, Hamalainen A, Al E. Natural history of the infant gut microbiome and impact of antibiotic treatments on strain-level diversity and stability. Sci Trans Med [Internet] 2016;8:1173–1178. Available from: 10.1126/scitranslmed.aad0917 10.1126/scitranslmed.aad0917 PMC5032909 27306663

[13] Gasparrini AJ, Wang B, Sun X, Kennedy EA, Hernandez-leyva A, Ndao IM, et al. Persistent metagenomic signatures of early-life hospitalization and antibiotic treatment in the infant gut microbiota and resistome. Nat Microbiol [Internet]. 2019;4:2285–2297. Available from: 10.1038/s41564-019-0550-2 10.1038/s41564-019-0550-2 PMC6879825 31501537

[14] Sosa-Moreno A, Comstock SS, Sugino KY, Ma TF, Paneth N, Davis Y, et al. Perinatal risk factors for fecal antibiotic resistance gene patterns in pregnant women and their infants. PLoS One [Internet]. 2020;15:1–21. Available from: 10.1371/journal.pone.0234751 10.1371/journal.pone.0234751 PMC7302573 32555719

[15] Bäckhed F, Roswall J, Peng Y, Feng Q, Jia H, Kovatcheva-Datchary P, et al. Dynamics and stabilization of the human gut microbiome during the first year of life. Cell Host Microbe [Internet]. 2015;17:690–703. Available from: 10.1016/j.chom.2015.04.004 10.1016/j.chom.2015.04.004 25974306

[16] Gosalbes MJ, Vallès Y, Jimenez-Hernandez N, Balle C, Riva P, Miravet-Verde S, et al. High frequencies of antibiotic resistance genes in infants’ meconium and early fecal samples. J Dev Orig Health Dis [Internet] 2016;7:35–44. Available from: 10.1017/S2040174415001506 10.1017/S2040174415001506 26353938

[17] Yassour M, Jason E, Hogstrom LJ, Arthur TD, Tripathi S, Siljander H, et al. Strain-level analysis of mother-to-child bacterial transmission during the first few months of life short article strain-level analysis of mother-to-child bacterial transmission during the first few months of life. Cell Host Microbe [Internet] 2018;24:146–154. Available from: 10.1016/j.chom.2018.06.007 10.1016/j.chom.2018.06.007 PMC6091882 30001517

[18] Moore AM, Ahmadi S, Patel S, Gibson MK, Wang B, Ndao IM, et al. Gut resistome development in healthy twin pairs in the first year of life. Microbiome [Internet]. 2015;3:1–10. Available from: 10.1186/s40168-015-0090-9 10.1186/s40168-015-0090-9 PMC4480905 26113976

[19] Pärnänen K, Karkman A, Hultman J, Lyra C, Bengtsson-palme J, Larsson DGJ (2018). Maternal gut and breast milk microbiota affect infant gut antibiotic resistome and mobile genetic elements. Nat Commun [Internet]..

[20] Shao Y, Forster SC, Tsaliki E, Vervier K, Strang A, Simpson N, et al. Stunted microbiota and opportunistic pathogen colonization in caesarean-section birth. Nature [Internet]. 2019;574:117–21. Available from: 10.1038/s41586-019-1560-1 10.1038/s41586-019-1560-1 PMC6894937 31534227

[21] Rahman SF, Olm MR, Morowitz MJ, Banfield JF. Machine Learning Leveraging Genomes from Metagenomes Identifies Influential Antibiotic Resistance Genes in the Infant Gut Microbiome. mSystems [Internet]. 2018;3:1–12. Available from: 10.1128/msystems.00123-17 10.1128/mSystems.00123-17 PMC5758725 29359195

[22] Esaiassen E, Hjerde E, Cavanagh JP, Pedersen T, Andresen JH, Rettedal SI, et al. Effects of probiotic supplementation on the gut microbiota and antibiotic Resistome development in preterm infants. Front Pediatr [internet]. 2018;6:1–16. Available from: 10.3389/fped.2018.00347. 10.3389/fped.2018.00347 PMC6250747 30505830

[23] Madan JC, Hoen AG, Lundgren SN, Farzan SF, Cottingham KL, Morrison HG, et al. Association of cesarean delivery and formula supplementation with the intestinal microbiome of 6-week-old infants. JAMA Pediatr [Internet] 2016;170:212–219. Available from: 10.1001/jamapediatrics.2015.3732 10.1001/jamapediatrics.2015.3732 PMC4783194 26752321

[24] Reyman M, van Houten MA, van Baarle D, Bosch AATM, Man WH, Chu MLJN, et al. Impact of delivery mode-associated gut microbiota dynamics on health in the first year of life. Nat Commun [internet]. Springer US; 2019;10:1–12. Available from: 10.1038/s41467-019-13014-7 10.1038/s41467-019-13014-7 PMC6825150 31676793

[25] Vatanen T, Plichta DR, Somani J, Münch PC, Arthur TD, Hall AB, et al. Genomic variation and strain-specific functional adaptation in the human gut microbiome during early life. Nat Microbiol [Internet]. 2019;4:470–479. Available from: 10.1038/s41564-018-0321-5 10.1038/s41564-018-0321-5 PMC6384140 30559407

[26] Coker MO, Hoen AG, Dade E, Lundgren S, Li Z, Wong AD, et al. Specific class of intrapartum antibiotics relates to maturation of the infant gut microbiota: a prospective cohort study. BJOG An Int J Obstet Gynaecol [Internet] 2019;127:217–227. Available from: 10.1111/1471-0528.15799 10.1111/1471-0528.15799 PMC6803026 31006170

[27] Azad MB, Konya T, Persaud RR, Guttman DS, Chari RS, Field CJ, et al. Impact of maternal intrapartum antibiotics, method of birth and breastfeeding on gut microbiota during the first year of life : a prospective cohort study. BJOG An Int J Obstet Gynaecol [Internet]. 2016;123:983–993. Available from: 10.1111/1471-0528.13601 10.1111/1471-0528.13601 26412384

[28] Mazzola G, Murphy K, Ross RP, Di Gioia D, Biavati B, Corvaglia LT, et al. Early gut microbiota perturbations following intrapartum antibiotic prophylaxis to prevent group B streptococcal disease. PLoS One [Internet] 2016;11:1–11. Available from: 10.1371/journal.pone.0157527 10.1371/journal.pone.0157527 PMC4917232 27332552

[29] Nogacka A, Salazar N, Suárez M, Milani C, Arboleya S, Solís G, et al. Impact of intrapartum antimicrobial prophylaxis upon the intestinal microbiota and the prevalence of antibiotic resistance genes in vaginally delivered full-term neonates. Microbiome [Internet]. 2017;5:1–10. Available from: 10.1186/s40168-017-0313-3 10.1186/s40168-017-0313-3 PMC5549288 28789705

[30] Vatanen T, Franzosa EA, Schwager R, Tripathi S, Arthur TD, Vehik K, et al. The human gut microbiome in early-onset type 1 diabetes from the TEDDY study. Nature [Internet]. 2018;562:589–594. Available from: 10.1038/s41586-018-0620-2 10.1038/s41586-018-0620-2 PMC6296767 30356183

[31] Yatsunenko T, Rey FE, Manary MJ, Trehan I, Dominguez-Bello MG, Contreras M, et al. Human gut microbiome viewed across age and geography. Nature [Internet]. 2012;486:222–7. Available from: https://www.nature.com/articles/nature11053.pdf 10.1038/nature11053 PMC3376388 22699611

[32] Korpela K, Salonen A, Saxen H, Nikkonen A, Peltola V, Jaakkola T, et al. Antibiotics in early life associate with specific gut microbiota signatures in a prospective longitudinal infant cohort. Pediatr res [internet]. Springer US; 2020;88:438–443. Available from: 10.1038/s41390-020-0761-5 10.1038/s41390-020-0761-5 31954376

[33] Stewart CJ, Ajami NJ, O’Brien JL, Hutchinson DS, Smith DP, Wong MC, et al. Temporal development of the gut microbiome in early childhood from the TEDDY study. Nature [Internet]. 2018;562:583–588. Available from: 10.1038/s41586-018-0617-x 10.1038/s41586-018-0617-x PMC6415775 30356187

[34] Kaminski J, Gibson MK, Franzosa EA, Segata N, Dantas G, Huttenhower C. High-Specificity Targeted Functional Profiling in Microbial Communities with ShortBRED. PLOS Comput Biol [Internet]. 2015;486:207–14. Available from: 10.1038/nature11234 10.1371/journal.pcbi.1004557 PMC4684307 26682918

[35] Alcock BP, Raphenya AR, Lau TTY, Tsang KK, Bouchard M, Edalatmand A, et al. CARD 2020: antibiotic resistome surveillance with the comprehensive antibiotic resistance database. Nucleic Acids Res [Internet]. 2020;48:D517–25. Available from: 10.1093/nar/gkz935 10.1093/nar/gkz935 PMC7145624 31665441

[36] Leng Z, Riley DE, Berger RE, Krieger JN, Roberts MC. Distribution and mobility of the tetracycline resistance determinant tetQ. J Antimicrob Chemother [Internet]. 1997;40:551–9. Available from: 10.1093/jac/40.4.551 10.1093/jac/40.4.551 9372425

[37] Shoemaker NB, Vlamakis H, Hayes K, Salyers AA. Evidence for extensive resistance gene transfer among Bacteroides spp. and among Bacteroides and other genera in the human colon. Appl Environ Microbiol [Internet]. 2001;67:561–8. Available from: https://aem.asm.org/content/67/2/561 10.1128/AEM.67.2.561-568.2001 PMC92621 11157217

[38] Segata N, Waldron L, Ballarini A, Narasimhan V, Jousson O, Huttenhower C. Metagenomic microbial community profiling using unique clade-specific marker genes. Nat Methods [Internet]. 2012;9:811–814. Available from: 10.1038/nmeth.2066 10.1038/nmeth.2066 PMC3443552 22688413

[39] Hu Y, Yang X, Qin J, Lu N, Cheng G, Wu N, et al. Metagenome-wide analysis of antibiotic resistance genes in a large cohort of human gut microbiota. Nat Commun [Internet] 2013;4:1–7. Available from: 10.1038/ncomms3151 10.1038/ncomms3151 23877117

[40] Casaburi G, Duar RM, Vance DP, Mitchell R, Contreras L, Frese SA, et al. Early-life gut microbiome modulation reduces the abundance of antibioticresistant bacteria. Antimicrob Resist Infect Control [Internet]. 2019;8:1–18. Available from: 10.1186/s13756-019-0583-6 10.1186/s13756-019-0583-6 PMC6693174 31423298

[41] Mallick H, McIver L, Rahnavard A, Ma S, Zhang Y, Nguyen LH, et al. Maaslin2: multivariable Association in Population-scale Meta-omics Studies [internet]. 2020. Available from: https://huttenhower.sph.harvard.edu/maaslin/10.1371/journal.pcbi.1009442 PMC8714082 34784344

[42] Scholz M, Ward D V., Pasolli E, Tolio T, Zolfo M, Asnicar F, et al. Strain-level microbial epidemiology and population genomics from shotgun metagenomics. Nat Methods [Internet]. 2016;13:435–438. Available from: 10.1038/nmeth.3802 10.1038/nmeth.3802 26999001

[43] Cao J, Hu Y, Liu F, Wang Y, Bi Y, Lv N, et al. Metagenomic analysis reveals the microbiome and resistome in migratory birds. Microbiome. 2020;8:1–18. 10.1186/s40168-019-0781-8 10.1186/s40168-019-0781-8 PMC7053137 32122398

[44] Shi P, Jia S, Zhang X-X, Zhang T, Cheng S, Li A. Metagenomic insights into chlorination effects on microbial antibiotic resistance in drinking water. Water res [internet]. Elsevier Ltd; 2013;47:111–120. Available from: 10.1016/j.watres.2012.09.046 10.1016/j.watres.2012.09.046 23084468

[45] Shin N-R, Whon TW, Bae J-W. Proteobacteria: microbial signature of dysbiosis in gut microbiota. Trends Biotechnol [internet]. Elsevier Ltd; 2015;33:496–503. Available from: 10.1016/j.tibtech.2015.06.011 10.1016/j.tibtech.2015.06.011 26210164

[46] Stecher B, Maier L, Hardt WD. “Blooming” in the gut: how dysbiosis might contribute to pathogen evolution. Nat Rev Microbiol. 2013;11:277–84. Available from: 10.1038/nrmicro2989 10.1038/nrmicro2989 23474681

[47] Doenhardt M, Seipolt B, Mense L, Winkler JL, Thürmer A, Rüdiger M, et al. Neonatal and young infant sepsis by group B streptococci and Escherichia coli: a single-center retrospective analysis in Germany—GBS screening implementation gaps and reduction in antibiotic resistance. Eur J Pediatr [Internet]. 2020;179:1769-1777. Available from: 10.1007/s00431-020-03659-8 10.1007/s00431-020-03659-8 PMC7547982 32447562

[48] Schrag SJ, Farley MM, Petit S, Reingold A, Weston EJ, Pondo T, et al. Epidemiology of invasive early-onset neonatal sepsis, 2005 to 2014. Pediatrics [Internet]. 2016;138:e20162013. Available from: 10.1542/peds.2016-2013 10.1542/peds.2016-2013 27940705

[49] Lozupone CA, Stombauch JI, Gordon JI, Jansson JK, Knight R. Diversity, stability and resilience of the human gut microbiota. Nature [Internet]. 2012;489:220–30. Available from: 10.1038/nature11550 10.1038/nature11550 PMC3577372 22972295

[50] Sinha T, Vich Vila A, Garmaeva S, Jankipersadsing SA, Imhann F, Collij V, et al. Analysis of 1135 gut metagenomes identifies sex-specific resistome profiles. Gut Microbes [Internet]. 2018;10:358–66. Available from: 10.1080/19490976.2018.1528822 10.1080/19490976.2018.1528822 PMC6546312 30373468

[51] Schröder W, Sommer H, Gladstone BP, Foschi F, Hellman J, Evengard B, et al. Gender differences in antibiotic prescribing in the community: a systematic review and meta-analysis. J Antimicrob Chemother [Internet]. 2016;71:1800–6. Available from: 10.1093/jac/dkw054 10.1093/jac/dkw054 27040304

[52] Smith DRM, Dolk FCK, Smieszek T, Robotham JV, Pouwels KB. Understanding the gender gap in antibiotic prescribing: a cross-sectional analysis of English primary care. BMJ Open [Internet]. 2018;8:1–7. Available from: 10.1136/bmjopen-2017-020203 PMC5855331 29472269

[53] Brooks AW, Priya S, Blekhman R, Bordenstein SR. Gut microbiota diversity across ethnicities in the United States. PLoS Biol [Internet]. 2018;16:1–24. Available from: 10.1371/journal.pbio.2006842 10.1371/journal.pbio.2006842 PMC6279019 30513082

[54] Martínez JL, Coque TM, Baquero F. What is a resistance gene? Ranking risk in resistomes. Nat Rev Microbiol [Internet]. 2015;13:116–123. Available from: 10.1038/nrmicro3399 10.1038/nrmicro3399 25534811

[55] Gilbert-Diamond D, Cottingham KL, Gruber JF, Punshon T, Sayarath V, Gandolfi AJ, et al. Rice consumption contributes to arsenic exposure in US women. Proc Natl Acad Sci USA [Internet]. 2011;108:20656–60. Available from: https://www.pnas.org/content/pnas/108/51/20656.full.pdf 10.1073/pnas.1109127108 PMC3251121 22143778

[56] Bolger AM, Lohse M, Usadel B. Trimmomatic : a flexible trimmer for Illumina sequence data. Bioinformatics [Internet]. 2014;30:2114–20. Available from:10.1093/bioinformatics/btu170 10.1093/bioinformatics/btu170 PMC4103590 24695404

[57] Franzosa EA, McIver LJ, Rahnavard G, Thompson LR, Schirmer M, Weingart G, et al. Species-level functional profiling of metagenomes and metatranscriptomes. Nat Methods [Internet] 2018;15:962–968. Available from: 10.1038/s41592-018-0176-y 10.1038/s41592-018-0176-y PMC6235447 30377376

[58] Huttenhower Lab. Mid-2017 comprehensive antibiotic resistance database (CARD) markers. [Internet]. 2017. Available from: https://huttenhower.sph.harvard.edu/shortbred

[59] McIver LJ, Abu-Ali G, Franzosa EA, Schwager R, Morgan XC, Waldron L, et al. BioBakery: a meta’omic analysis environment. Bioinformatics [Internet].2018;34:1235–7. Available from: 10.1093/bioinformatics/btx754 10.1093/bioinformatics/btx754 PMC6030947 29194469

[60] Lloyd-Price J, Arze C, Ananthakrishnan AN, Schirmer M, Avila-Pacheco J, Poon TW, et al. Multi-omics of the gut microbial ecosystem in inflammatory bowel diseases. Nature [Internet] 2019;569:655–662. Available from: 10.1038/s41586-019-1237-9 10.1038/s41586-019-1237-9 PMC6650278 31142855

[61] Fong Y, Huang Y, Gilbert PB, Permar SR. Chngpt: threshold regression model estimation and inference. BMC Bioinformatics [Internet]. 2017;18:1–7.Available from: 10.1186/s12859-017-1863-x 10.1186/s12859-017-1863-x PMC5644082 29037149

[62] McMurdie PJ, Holmes S. Phyloseq: an R package for reproducible interactive analysis and graphics of microbiome census data. PLoS One [Internet]. 2013;8:e61217. Available from: 10.1371/journal.pone.0061217 10.1371/journal.pone.0061217 PMC3632530 23630581

[63] Lahti L, Sudarshan S. Tools for microbiome analysis in R. [Internet]. 2017. Available from: https://microbiome.github.io/tutorials/

[64] Gloor GB, Macklaim JM, Pawlowsky-Glahn V, Egozcue JJ. Microbiome datasets are compositional: and this is not optional. Front Microbiol [Internet] 2017;8:1–6. Available from: 10.3389/fmicb.2017.02224 10.3389/fmicb.2017.02224 PMC5695134 29187837

[65] Oksanen J, Blanchet FG, Friendly M, Kindt R, Legendre P, McGlinn D, et al. vegan: Community Ecology Package. R package version 2.5-6. [Internet]. 2019.

[66] Kolde R. pheatmaps: Pretty Heatmaps. R package version 2.5-6. [Internet]. 2019.

[67] Maamar S. Ben, Glawe AJ, Brown TK, Hellgeth N, Hu J, Wang J-P, et al. Mobilizable antibiotic resistance genes are present in dust microbial communities. PLoS Pathog [Internet]. 2020;16:e1008211. Available from: 10.1371/journal.ppat.1008211 10.1371/journal.ppat.1008211 PMC6977718 31971995

