# Supplementary material for: The infant gut resistome is associated with E. coli and early-life exposures
Source: BMC Microbiol. 2021 Jul 2;21:201. doi: 10.1186/s12866-021-02129-x (PMC8252198; doi:10.1186/s12866-021-02129-x)
Supplement: Supplementary file 1 — Additional file 1 Fig. S1: Relative abundance and distribution of number of unique antimicrobial resistance gene markers by sample age. Fig. S2: Longitudinal change in relative abundance of ARG markers colored by mechanism of antibiotic resistance (antibiotic efflux or not). Fig. S3: Heat map showing the most prevalent antibiotic resistance genes (present in at least 60% of samples) by sample. Fig. S4: Differential unique antimicrobial resistance genes and E. coli relative abundance between paired samples by delivery mode. Fig. S5: E. coli strains do not have differential genes based on early-life exposures. Fig. S6: Four gene families related to mobile genetic elements are associated with sample age and specific taxa. Fig. S7: Overview of Analytic Steps and Main Statistical Methods. Fig. S8: Quality control to assess the validity of the sequencing and downstream analysis tools. Supplementary Methods: Infant Feeding Mode up to the Time of Stool Sample Collection. [file 12866_2021_2129_MOESM1_ESM.docx]

**The Infant Gut Resistome is Associated with *E. coli* and Early-life Exposures**

Rebecca M. Lebeaux, BS, Modupe O. Coker, BDS, MPH, PhD, Erika F. Dade, MS, Thomas J. Palys, PhD, Hilary G. Morrison, PhD, Benjamin D. Ross, PhD, Emily R. Baker, MD, Margaret R. Karagas, PhD, Juliette C. Madan, MS, MS, Anne G. Hoen, PhD

Table of Contents

**Figure S1:** Relative abundance and distribution of number of unique antimicrobial resistance gene markers by sample age………………………………………………..……2

**Figure S2:** Longitudinal change in relative abundance of ARG markers colored by mechanism of antibiotic resistance (antibiotic efflux or not)………………………………..3

**Figure S3:** Heat map showing the most prevalent antibiotic resistance genes (present in at least 60% of samples) by sample………………………………………………………..4

**Figure S4:** Differential unique antimicrobial resistance genes and *E. coli* relative abundance between paired samples by delivery mode…………………………………….5

**Figure S5:** *E. coli* strains do not have differential genes based on early-life exposures..6

**Figure S6:** Four gene families related to mobile genetic elements are associated with sample age and specific taxa……………………………………………………………....….7

**Figure S7:** Overview of Analytic Steps and Main Statistical Methods…………………….8

**Figure S8:** Quality control to assess the validity of the sequencing and downstream analysis tools…………………………………………………………………………………….9

**Supplementary Methods**: Infant Feeding Mode up to the Time of Stool Sample Collection…………………………………………………………………………..……….10-11


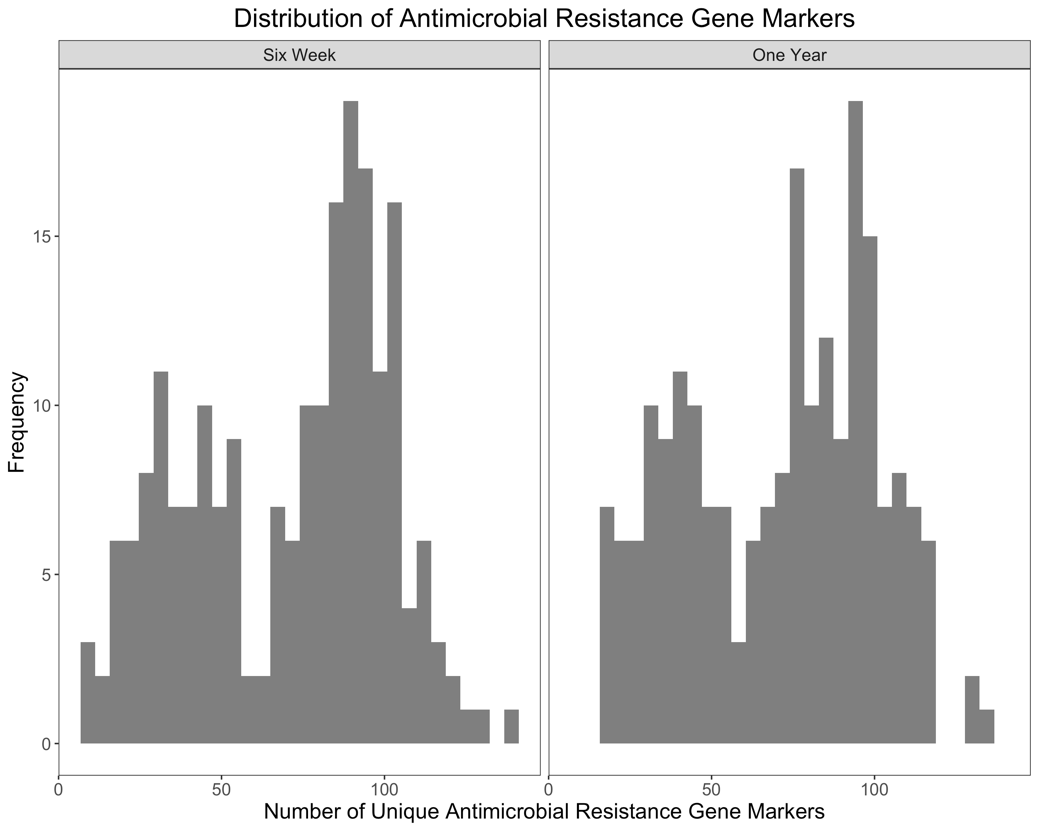


a
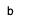


b
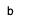


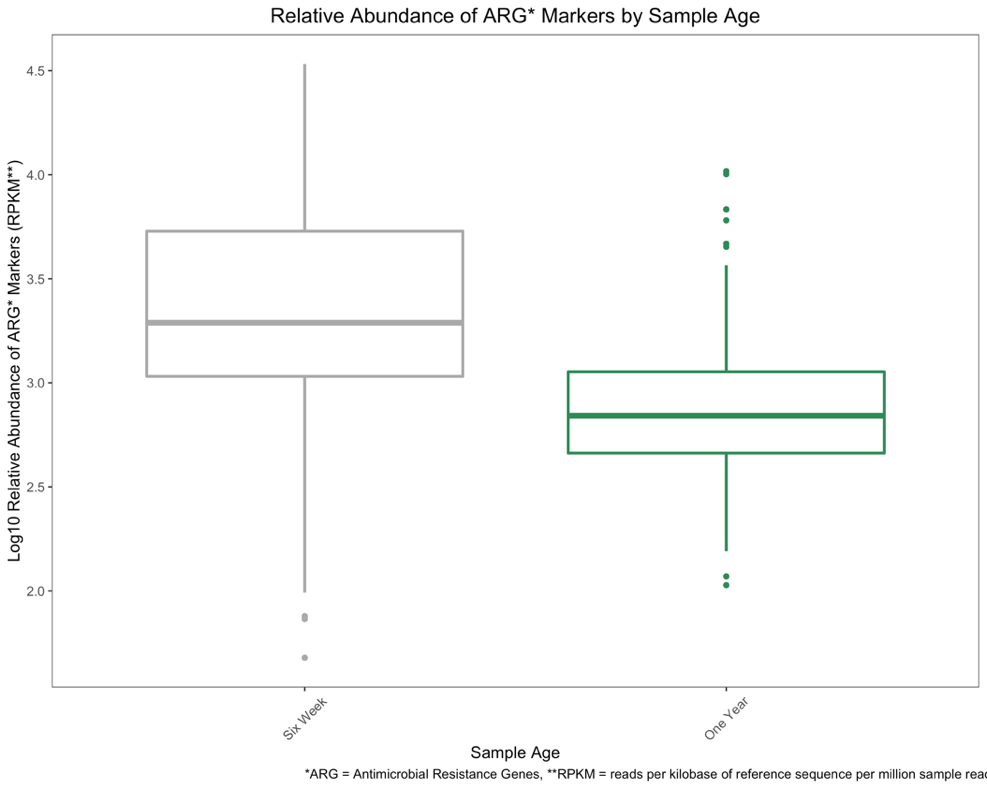


*p*-value < 0.01

**Figure S1:** Relative abundance and distribution of number of unique antimicrobial resistance gene markers by sample age. (a) Distribution of unique antimicrobial resistance gene markers stratified by sample age. (b) Log_10_ relative abundance of overall antimicrobial resistance gene markers in RPKM. Difference in mean RPKM between two sample age time points has a *p*-value < 0.01 using a Wilcoxon rank sum test. *ARG = antimicrobial resistance genes, **RPKM = reads per kilobase of reference sequence per million sample reads


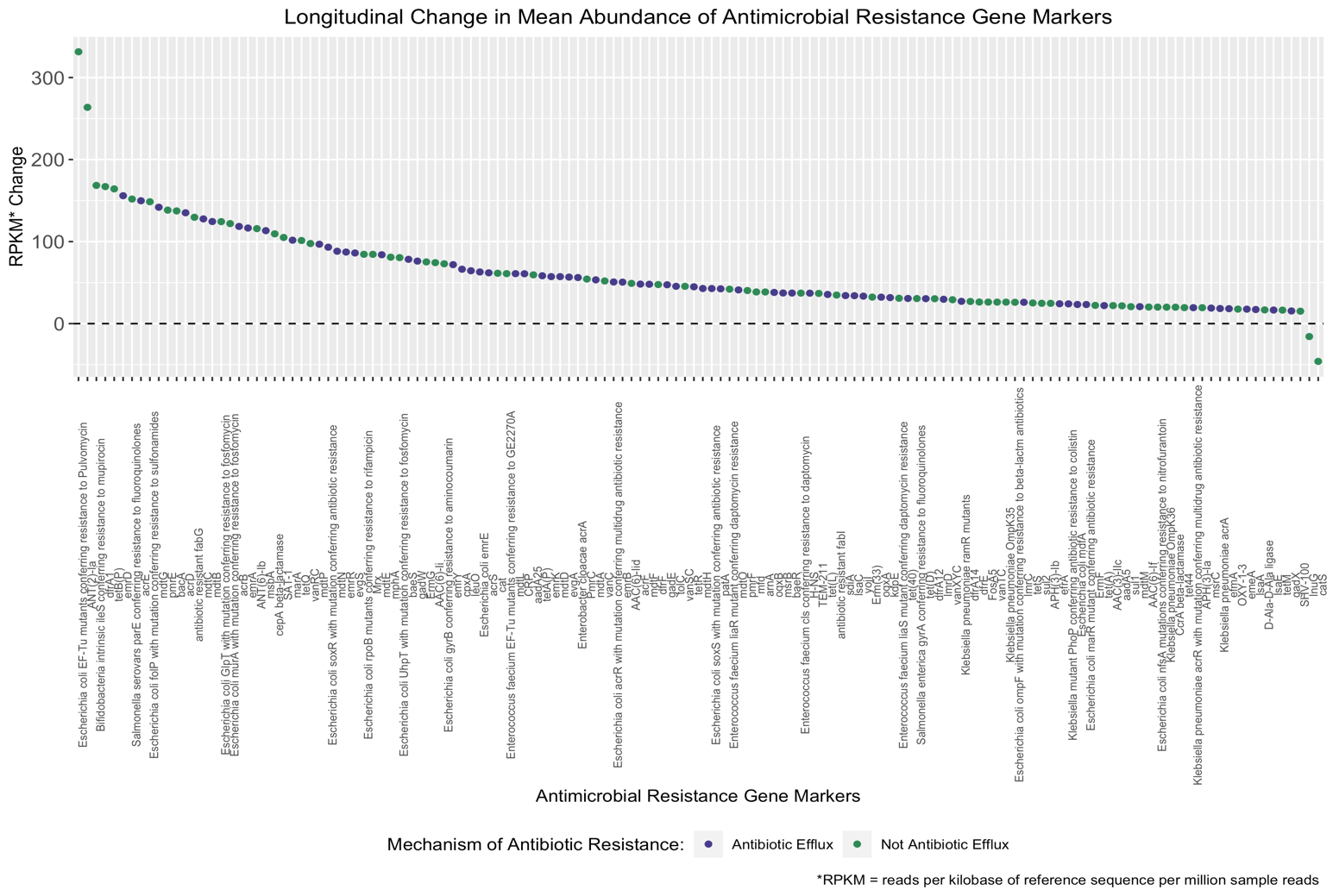


**Figure S2:** Longitudinal change in relative abundance of antimicrobial resistance gene (ARG) markers colored by mechanism of antibiotic resistance (antibiotic efflux or not). Only genes that had a net relative abundance change of 15 RPKM or greater are plotted.

> 6

weeks

> 1

year

*RPKM Change

**Figure S3**: Heat map showing the most prevalent antibiotic resistance genes (present in at least 60% of samples) by sample. ARG (*x*-axis) presence/absence clustered by specific features of the samples (*y*-axis) including sample age, *E. coli* relative abundance, Proteobacteria relative abundance, delivery mode, and the number of unique ARGs per sample. ARGs and samples are clustered using Euclidean distance. ARG presence is indicated if the ARG was in a RPKM relative abundance of at least 0.01.


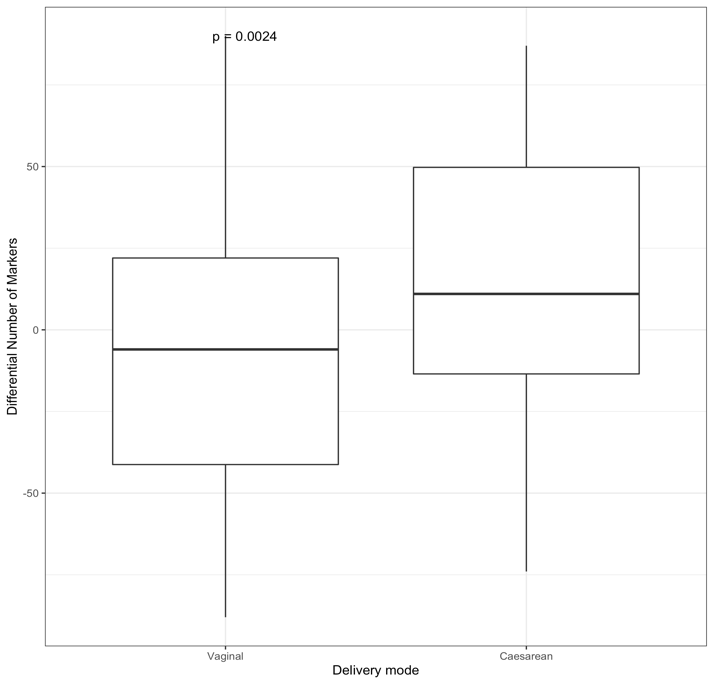

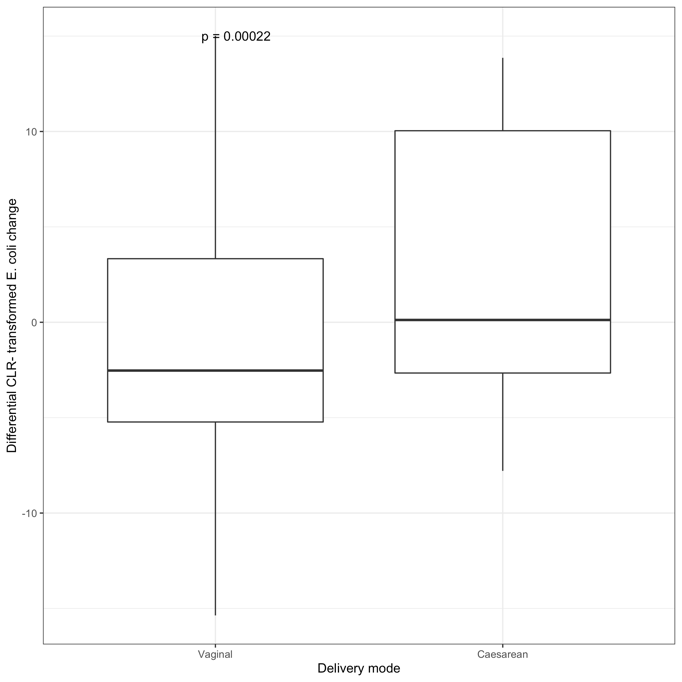


p-value < 0.01

b

a

p-value < 0.01

**Figure S4**: Differential unique antimicrobial resistance genes and *E. coli* relative abundance between paired samples by delivery mode. (a) Differential number of antimicrobial resistance gene markers from six weeks to one year in infants delivered vaginally compared to by c-section. (b) Differential centered log-ratio transformed relative abundance of *E. coli* by delivery mode. Difference in mean between two methods of delivery mode have *p*-value < 0.01 tested using a Wilcoxon rank sum test.

a

b


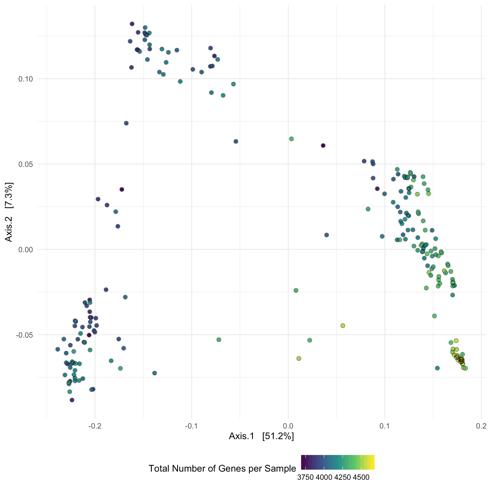


**Figure S5**: *E. coli* strains do not have differential genes based on early-life exposures. (a) Multidimensional scaling of gene presence/absence matrix using the jaccard distance metric of dominant strain of *E. coli* clustered by the number of markers by samples. (b) Heat map showing the 20-80% of genes prevalent in each sample’s dominant *E. coli.* Both rows and columns are clustered using correlation. Green indicates gene presence and blue indicates gene absence.


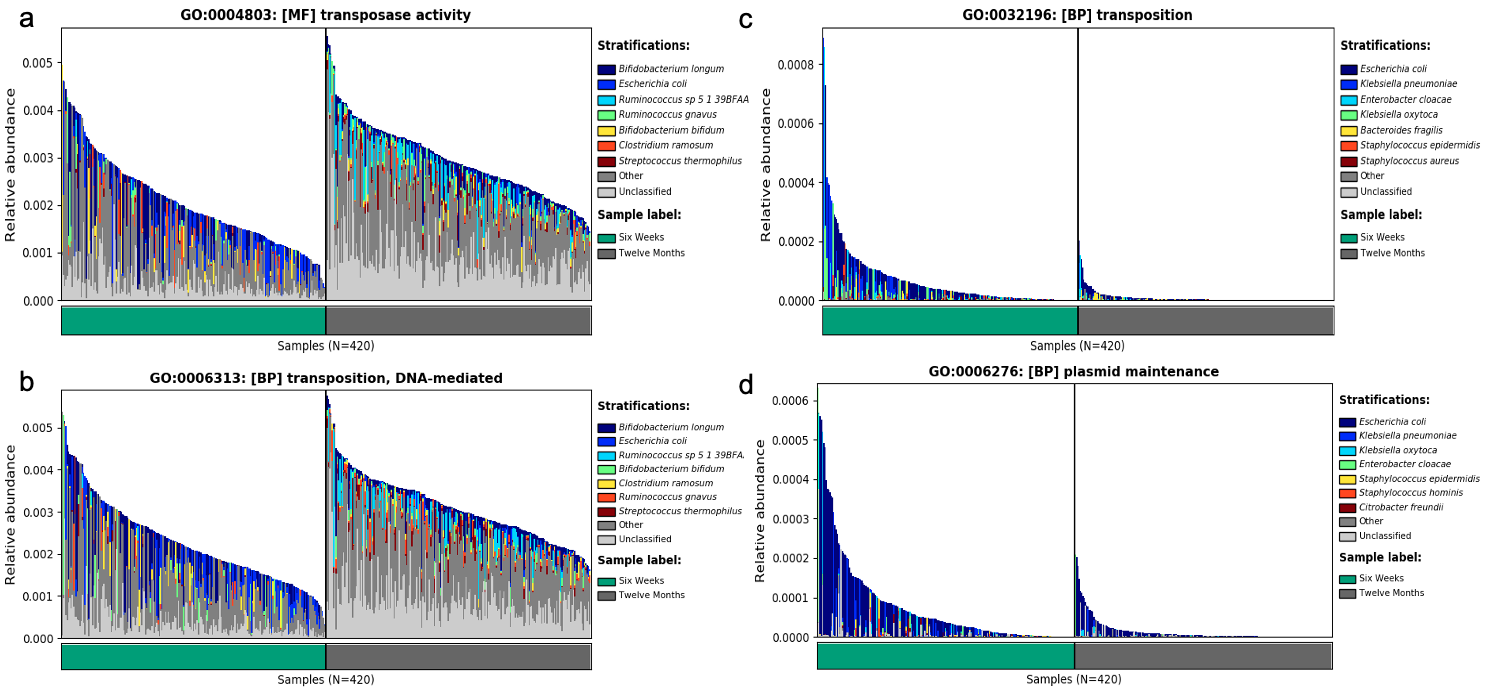


**Figure S6:** Four gene families related to mobile genetic elements are associated with sample age and specific taxa. Two of these gene families (transposase activity and transposition, DNA-mediated) had a higher overall relative abundance at 1 year compared to at 6 weeks based on MaAsLin2 (a and b) while transposition and plasmid maintenance (c and d) were at a higher abundance at 6 weeks. In all of the gene families, species-specific contributions by *E. coli* relative abundance appears to be a major contributing factor to the association.

**Figure S7**: Overview of analytic steps and main statistical methods. This diagram outlines the main steps taken in this analysis. Steps were conducted from top to bottom. The following profiling tools were used: ShortBRED for ARGs, MetaPhlAn2 for taxonomy, PanPhlAn for strain analysis of *E. coli*, and HUMAnN2 for the mobile genetic elements analysis. ARG = antimicrobial resistance gene


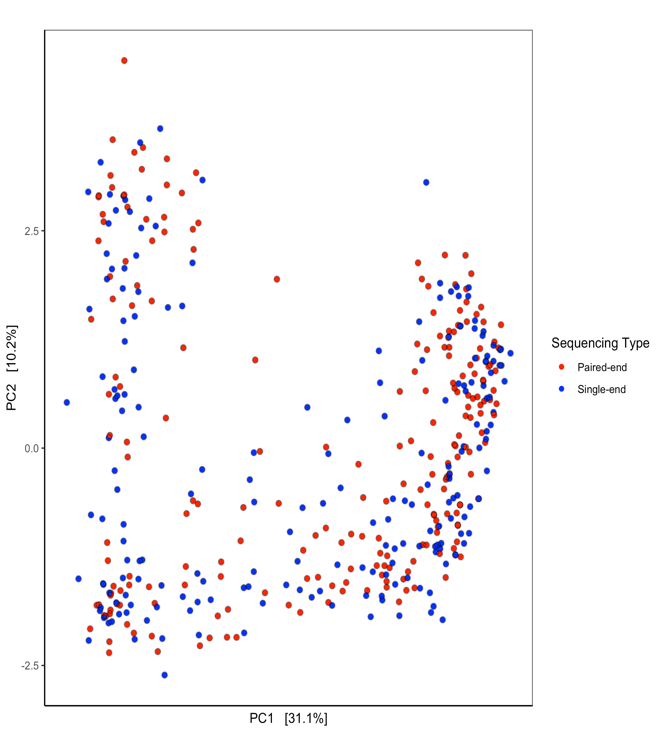

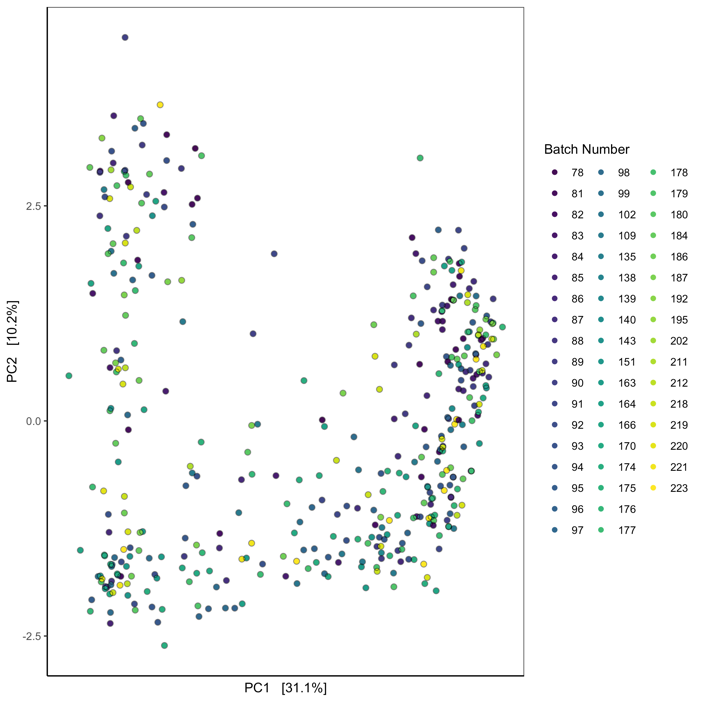
**
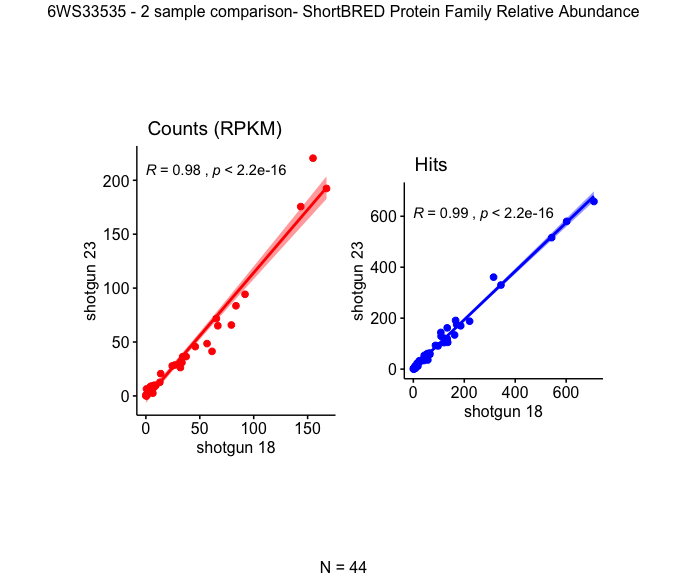

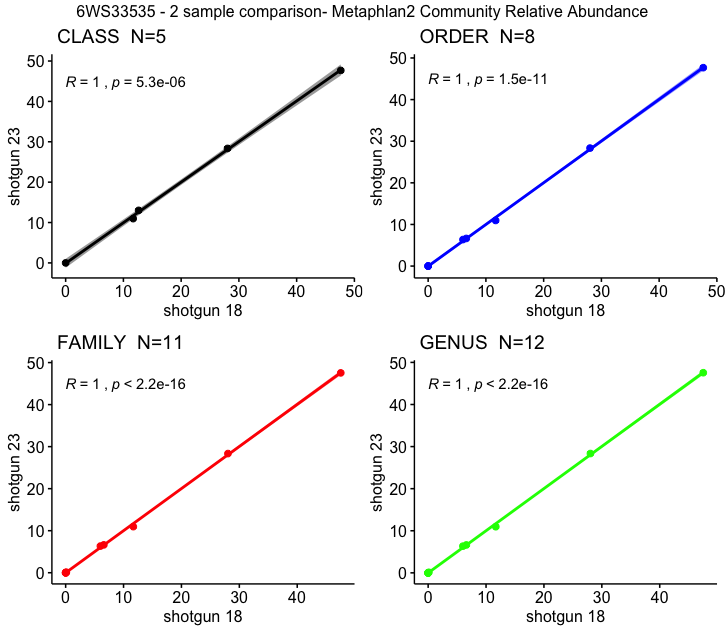
**

b

d

a

c

**Figure S8**: Quality control to assess the validity of the sequencing and downstream analysis tools. (a) and (b) Principal Component Analysis of the resistome. The relative abundance of resistome samples have been centered log-ratio transformed and have been plotted using the Euclidean distance. (a) shows the samples by sequencing type (paired or single-end) and (b) is by sequencing batch number. (c) and (d) Downstream analysis of duplicated 6-week sample with shotgun 18 and 23 numbered for the batches the sample was run on. *P*-values calculated using pearson correlation. (c) Counts are the normalized count for the antimicrobial resistance gene marker in reads per kilobase of reference sequence per million sample reads and hits is the number of valid hits to the antimicrobial resistance gene marker. (d) MetaPhlAn2 relative abundance overlap between the duplicated sample runs.

**Supplementary Methods:** Infant Feeding Mode up to the Time of Stool Sample Collection*

Description:

Infant feeding mode is a variable derived from the New Hampshire Birth Cohort Study (NHBCS) covariate data. It is a function of sample age and it was inferred from questionnaire data that could include time periods from 4, 8, 12, 18, 24, 30, 36, 42, 48, 54, and 60 months, as well as the age of breastmilk samples if present for a mother-infant dyad.

Feeding mode up to the time of stool sample collection was defined as:

0 = unable to determine from available information

1 = exclusively breastfed

2 = exclusively formula fed

3 = combination fed

The feeding mode censor is defined as:

0 = zero or little confidence in inferred feeding mode value; use not recommended

1 = reasonable confidence based on the information available

Method:

The custom code computes feeding mode up to the time of collection of individual stool samples and appends any new samples to pre-existing sample records. A key contribution is the software driven interactive analysis of formula text derived from telephone questionnaires to determine if the entry represents formula, milk, substitute breastmilk, or other non-formula foods. These entries are catalogued in a formula dictionary and aid in the detection of non-formula entries that would otherwise be interpreted as formula fed to the infant. The number of unique entries in the formula dictionary increases as new text is encountered. Every iteration of the script preserves previous calculations that have a censor = 1, replaces calculations that have a censor = 0, and makes suggested edits if new information has resulted in a different feeding mode suggestion. Every entry is reviewed by a data analyst with particular attention to infants that the script flags as potentially having conflicting information. The feeding mode censor is set to 0 when there are unresolvable discrepancies in the questionaire responses.

The telephone questionnaire data contained questions related to formula use based on responses at three possible time points: at 4, 8, and 12 months. Additional timepoints beyond the 12 month time point are used to augment breastfeeding history. Presence of breastmilk samples is also used to corroborate a minimum age of last breastfeeding. Infant stool samples collected at ages approximately 6 weeks and 12 months do not necessarily coincide with the timing of questionnaire responses. Therefore, two assumptions were made to ground the ultimate classification of feeding mode up to the time of sample collection: 1) a self-consistent telephone interview response with an interview age that is older than but nearest in time to the sample age is assumed to be more accurate than a later recall; and 2) it is assumed that infants are breastfed from birth if at all.

All samples with a feeding mode or censor of 0 were removed from this analysis.

*If the age of collection is not available, the age of receipt is used.
